# Disease-induced changes in *Panax ginseng* phyllosphere fungal community assembly and functional adaptation

**DOI:** 10.3389/fmicb.2026.1740520

**Published:** 2026-03-26

**Authors:** Liu Shuang, Tian Ge, Liu Hang, Li Wenjing, Xu Jiang, Hu Haoyu, Xiao Shuiming

**Affiliations:** 1 Houji Laboratory in Shanxi Province, Shanxi Institute for Functional Food, Shanxi Agricultural University, Taiyuan, China; 2 Key Laboratory of Minor Crop Germplasm Innovation and Molecular Breeding (Co-construction by Ministry and Province), Ministry of Agriculture and Rural Affairs, Taiyuan, China; 3 State Key Laboratory for Quality Ensurance and Sustainable Use of Dao-Di Herbs, Institute of Chinese Materia Medica, China Academy of Chinese Medical Sciences, Beijing, China

**Keywords:** biocontrol mechanism, fungi, infectious diseases, *Panax ginseng*, phyllosphere microbiota

## Abstract

**Introduction:**

Phyllosphere microorganisms play essential roles in plant health and disease resistance, yet their responses to pathogen infections remain poorly understood. *Panax ginseng* is susceptible to multiple fungal diseases, which threaten its quality and yield. This study aimed to clarify the underlying disease resistance mechanisms of *Panax ginseng* by analyzing the phyllosphere fungal communities associated with fungal infections.

**Methods:**

Phyllosphere fungal communities of healthy *Panax ginseng* plants and those with three fungal infections (gray mold, damping-off and root rot) were compared to explore the disease resistance mechanisms related to fungal community changes.

**Results:**

Results revealed distinct niche differentiation: leaves were dominated by Basidiomycota (82.0%), while stems harbored more Ascomycota (94.2%), including pathogens like *Monilinia laxa* (35.73%). Fungal infection significantly reduced microbial alpha diversity, altered community structure (PERMANOVA, *p* = 0.001), and destabilized co-occurrence networks (modularity decreased from 0.8501 to 0.8116). Functional prediction indicated downregulation of key metabolic pathways (e.g., NAD/NADP interconversion, phospholipid biosynthesis). Disease stress induced an enrichment of potentially beneficial taxa (e.g., *Rhodotorula*) in leaves, indicative of a limited antagonistic response, while the overall community was ultimately dominated and disrupted by pathogens.

**Discussion:**

Elucidating these compositional shifts of phyllosphere fungal communities advances the understanding of plant–microbe–pathogen interactions and provides a critical theoretical groundwork for development of microbiome-driven early disease diagnosis, resistance breeding, and eco-friendly disease control strategies for *Panax ginseng*.

## Introduction

The aboveground parts of terrestrial plants, collectively known as the phyllosphere, are normally inhabited by a diverse community of bacteria, fungi, yeasts and actinomycetes (Lindow and Brandl, 2003). Some of these microorganisms occur as epiphytes on plant surfaces, whereas others reside inside leaves as endophytes (Beattie and Lindow, 1999). The interactions between plant and phyllosphere microbiota play essential roles in host functionality and fitness, including nutrient acquisition, abiotic stress tolerance, and disease suppression (Stone et al., 2018; Sohrabi et al., 2023). Although the phyllosphere may be colonized by pathogenic microorganisms, its resident microbiota actively contributes to plant disease resistance (Tao et al., 2023). However, in contrast to the well-characterized functions of root-colonizing microbiota in plant health (Berendsen et al., 2012; Hacquard et al., 2015; Durán et al., 2018), the collective community-level contributions of phyllosphere microbiota to plant growth, development, and health remain poorly understood.

*Panax ginseng* C. A. Mey., a deciduous perennial plant belonging to the Araliaceae family, has been used for millennia as a medicinal tonic or adaptogenic agent, and is now widely incorporated in functional foods, cosmetics, and beverages (Xu et al., 2017). The global value of *P. ginseng* is now estimated at 2.0 and 3.5 billion dollars per year. China accounts for approximately 80% of global ginseng yield, mainly supplied by cultivated alternatives (Jung et al., 2003; Hong et al., 2006; Ying et al., 2012; Baeg and So, 2013; Dong et al., 2018). Ginseng, as a medicinal herb and food resource, is vulnerable to infection by diverse foliar and soil-borne pathogens during their growth cycle. In the absence of human intervention, its final survival rate is below 25% (Li et al., 2014; Dong et al., 2018). To date, over 10 major diseases have been reported in *P. ginseng*, causing symptoms such as gray mold, damping-off, root rot, sudden wilt, black spot, sclerotinia stem rot, phytophthora blight, rust rot, etc., some of which are destructive (Lee et al., 2011; Gao et al., 2014; Wang et al., 2016; Farh et al., 2018; Bischoff Nunes and Goodwin, 2022).

Although advances have been achieved in the integrated control of ginseng infectious diseases, the application of chemical pesticides (e.g., fludioxonil, hymexazol, and thiophanate-methyl) remains an a primary approach of chemical control, causing their overuse and misuse. The rational use of microbial-derived biocontrol agents can effectively reduce the use of chemical fertilizers and pesticides, enhance crop yield and quality, while protecting the ecological environment (Mitter et al., 2019).

However, the screening of most biocontrol bacteria/fungi remains confined to the laboratory stage. During field application, several challenges persist such as high susceptibility to the environmental influences, difficulty in colonization, unstable activity, and insignificant field efficacy of single strains. Studies have shown that the endophytic and rhizosphere microorganisms of ginseng are invloved to varying degrees in the interaction network among ginseng, biocontrol agents, and pathogens, indicating that research on biocontrol agents should expand from a single-strain focus to microbial consortia (Vogel et al., 2021).

Based on the description of the structure and biological functions of the ginseng phyllosphere microbiota, a deeper understanding of its complexity and the interaction network among ginseng, the microbiota, and pathogens will provide novel research insights into the mechanisms underlying the prevention and control of ginseng infectious diseases, as well as the development of more scientific prevention and control strategies (Trivedi et al., 2020; Vogel et al., 2021). Recent studies have demonstrated that microbial agents, such as *Paenibacillus mucilaginosus* and *Bacillus subtilis*, can reshape the rhizosphere microbiome, enhance the accumulation of bioactive compounds, and improve plant growth in medicinal plants like *Epimedium pubescens* (Lai et al., 2025). The three fungal diseases—gray mold, damping-off, and root rot—differ substantially in their infection sites, pathogenic strategies, and life histories. By integrating these pathosystems into a unified analytical framework, this study aimed to address the following questions: (1) how infection site modulates the response patterns of the aboveground microbiome; (2) whether different pathogens drive convergent or divergent successional trajectories in the microbiome; and (3) how the stem-associated microbiome, as an interface between above- and belowground compartments, responds to stress originating from distinct sources. From a comparative pathology perspective, this work not only elucidates the microbiome-mediated mechanisms underlying individual diseases, but also aims to extract common micro-ecological principles regulating the response of *P. ginseng* to fungal stress. The findings provide a theoretical foundation for developing microbiome-based, broad-spectrum yet precise ecological strategies for disease management.

## Materials and methods

### Plant materials

Fresh stem and leaf samples were collected from 4- and 5-year-old *P. ginseng* plant at Baishan Lincun Medicine Development Co., Ltd. in Jingyu County of Jilin Province, China (N42°21′18.85″, E126°45′44.94″). These tissue samples were obtained from ginseng plants cultivated in a planting base, which was managed according to the Good Agricultural Practices (GAP) and the Specification on Good Agriculture and Collection Practices for Medicinal Plants (GACP). A total of 88 samples were collected. Based on the typical disease symptoms observed on ginseng leaves and stems (Supplementary Figure S1), three fungal infectious diseases were preliminarily identified and assigned the following group codes: GM (gray mold; *n* = 24), DO (damping-off; *n* = 22), and RR (root rot; *n* = 22), along with a healthy control group (CON; *n* = 20). All the samples were immediately frozen in liquid nitrogen and subsequently transferred for experimental analysis.

### DNA extraction

Approximately 5.0 g of samples were taken and rinsed briefly with sterile water to remove surface debris. The samples were then ground into powder using liquid nitrogen, and then lysis buffer containing lysozyme and proteinase K was added. The resulting mixture was incubated at 37 °C for 30 min to disrupt the cell walls and simultaneously extract total DNA from both surface-associated and endophytic microorganisms. Total genomic DNA samples were extracted using the OMEGA Soil DNA Kit (Cat: D5625-01, Omega Bio-Tek, GA, United States), following the manufacturer’s guidelines. The DNA samples were then stored at −20 °C for further analysis. The quality and integrity of the extracted DNA was assessed using a NanoDrop ND-1000 spectrophotometer (Thermo Fisher Scientific, Waltham, MA, United States) and agarose gel electrophoresis, respectively.

### ITS amplicon sequencing

PCR amplification of the fungal ITS1(b) region was performed using the forward primer ITS1F (5′-CTTGGTCATTTAGAGGAAGTAA-3′) and the reverse primer ITS2 (5′-GCTGCGTTCTTCATCGATGC-3′). Sample-specific, 7-bp barcodes were incorporated into the primers for multiplex sequencing. The PCR components contained 5 μL of buffer (5×), 0.25 μL of Fast pfu DNA Polymerase (5 U/μl), 2 μL (2.5 mM) of dNTPs, 1 μL (10 μM) of each Forward and Reverse primer, 1 μL of DNA Template, and 14.75 μL of ddH2O. Thermal cycling consisted of initial denaturation at 98 °C for 5 min, followed by 28 cycles consisting of denaturation at 98 °C for 30 s, annealing at 55 °C for 30 s, and extension at 72 °C for 45 s, with a final extension of 5 min at 72 °C. PCR amplicons were purified using Vazyme VAHTSTM DNA Clean Beads (Vazyme, Nanjing, China) and quantified using the Quant-iT PicoGreen dsDNA Assay Kit (Invitrogen, Carlsbad, CA, United States). After the individual quantification step, amplicons were pooled in equal amounts, and paired-end 2 × 250 bp sequencing was performed using the Illumina MiSeq platform with MiSeq Reagent Kit v3 at Shanghai Personal Biotechnology Co., Ltd. (Shanghai, China).

### Sequence analysis

The microbiome bioinformatics were performed using QIIME2 2019.4 (Bolyen et al., 2019) with slight modifications according to official tutorials.^1^ Briefly, raw sequence data were demultiplexed using the demux plugin following by primer cutting with cutadapt plugin (Martin, 2011). Sequences were then quality filtered, denoised, merged, and chimera were removed using the DADA2 plugin (Callahan et al., 2016). Non-singleton amplicon sequence variants (ASVs) were aligned with MAFFT (Katoh et al., 2002) and used to construct a phylogeny with FastTree2 (Price et al., 2010). The α-diversity metrics (Chao1, Observed species, Shannon, Simpson, etc.), and the β-diversity metrics (weighted/unweighted UniFrac, Jaccard distance, and Bray–Curtis dissimilarity) were estimated using the diversity plugin with samples rarefied to 58,144 fungal sequences per sample. Taxonomy was assigned to ASVs using the classify-sklearn naïve Bayes taxonomy classifier in the feature-classifier plugin (Bokulich et al., 2018) against the UNITE Release 8.0 (Fungi) Database (Kõljalg et al., 2013).

### Bioinformatics and statistical analysis

Sequence data analyses were mainly performed using QIIME2 and R packages (v3.2.0). ASV-level α-diversity indices were calculated using the ASV table in QIIME2, and visualized as box plots. ASV-level ranked abundance curves were generated to compare the richness and evenness of ASVs among samples. The β-diversity analysis was performed to investigate the structural variation of microbial communities across samples using Jaccard, Bray-Curtis and UniFrac distance metrics and visualized *via* principal coordinate analysis (PCoA), nonmetric multidimensional scaling (NMDS) and unweighted pair-group method with arithmetic means (UPGMA) hierarchical clustering. Principal Component Analysis (PCA) was also conducted based on the genus-level compositional profiles. The significance of differentiation of microbiota structure among groups was assessed by PERMANOVA (Permutational Multivariate Analysis of variance), ANOSIM (Analysis of Similarities) and Permdisp using QIIME2. The taxonomic compositions and abundances were visualized using MEGAN (Huson et al., 2011) and GraPhlAn (Asnicar et al., 2015). Venn diagram was generated to visualize the shared and unique ASVs among samples or groups using the R package “VennDiagram,” based on the occurrence of ASVs across samples/groups regardless of their relative abundance (Zaura et al., 2009). Taxa abundances at the ASV level were statistically compared among samples or groups by MetagenomeSeq, and visualized as Manhattan plots. LEfSe (Linear Discriminant Analysis Effect Size) was performed to detect differentially abundant taxa across groups using the default parameters (Segata et al., 2011). Random Forest analysis was applied to discriminate the samples from different groups using QIIME2 with default settings (Liaw and Wiener, 2002). Nested stratified k-fold cross validation was used for automated hyperparameter optimization and sample prediction. The number of K-fold cross-validations was set to 10. Co-occurrence network analysis was performed using SparCC analysis. The pseudocount value in SparCC was set to 10^−6^. The cutoff of correlation coefficients was determined as 70 through random matrix theory-based methods as implemented in the R package RMThreshold. Based on the correlation coefficients, we constructed a co-occurrence network, with nodes representing OTUs and edges representing correlations between these OTUs. The network was visualized using the R packages igraph and ggraph. Microbial functions were predicted by PICRUSt2 (Phylogenetic investigation of communities by reconstruction of unobserved states) using the MetaCyc^2^ and KEGG^3^ databases.

## Results

### Ecological niche and fungal infectious diseases affect ginseng phyllosphere microbiota assembly

After primer removal, quality filtering, denoising, sequence assembly, and chimera removal, a total of 102,090 ± 17,137 high-quality sequences were obtained per sample. When samples were grouped by ecological niche (leaf *vs*. stem), the mean sequence counts were 100,670 ± 19,889 (leaves) and 103,874 ± 12,916 (stems), with no significant difference in sequencing depth (*p* = 0.38666). For disease type-based groupings, one-way ANOVA revealed a significant overall difference (Overall ANOVA *p* = 0.03149). *Post-hoc* Tukey tests for mean comparisons identified that the RR group had a significantly higher sequence count than the CON group (Tukey test, *p* = 0.0358). However, infectious plant diseases were found not to significantly affect the microbiome sequencing depth observed in leaf-associated microbial communities (Overall ANOVA *p* = 0.47199). However, in the stem habitat, the GM, RR, and DO groups all displayed significantly higher sequence counts than the CON group (Supplementary Table S1). Sequence length distribution primarily occurred between 209 and 231 bp, accounting for 83.63% of the total sequences (Supplementary Figure S2).

An analysis of the taxonomic composition of fungal communities based on ASV tables revealed distinct microbial distribution patterns between leaf and stem niches. At the phylum level, leaves were predominantly colonized by *Basidiomycota* (82.0%), while *Ascomycota* accounted for just 8.92%. In contrast, stems exhibited an inverse pattern: *Ascomycota* dominated with 94.2% of the total, while *Basidiomycota* was reduced sharply to just 2.93% (Supplementary Figure S3A). At the genus level, the leaves were characterized by high abundances of *Rhodotorula* (45.98%), *Vishniacozyma* (20.03%), and *Filobasidium* (6.32%), while the stems showed higher levels of *Monilinia* (35.73%), *Plectosphaerella* (22.95%), and *Fusarium* (6.08%) (Supplementary Figures S3B,E). At the species level, the dominant taxa in the leaves were *Vishniacozyma victoriae* (17.67%), *Filobasidium magnum* (6.29%), and *Vishniacozyma carnescens* (2.04%), while the stems showed higher abundances of potential plant pathogens, such as *Monilinia laxa* (35.73%), *Fusarium solani* (2.30%), and *Cadophora luteo-olivacea* (1.49%), as well as other taxa including *Trichoderma hamatum* (2.71%) and *Cadophora orchidicola* (2.54%) (Supplementary Table S2). Collectively, these results indicate that stems are colonized by potential phytopathogens, such as *F. solani* and *M. laxa*, whereas leaves lack significant pathogen enrichment but harbor putatively beneficial yeasts, such as *Vishniacozyma* spp. In order to clarify whether these beneficial taxa are inherently present in healthy leaves or are “recruited” in response to a pathogen challenge, comparative analyses between diseased and healthy control groups are required.

When grouped by infectious disease, the overall composition (Supplementary Figure S3C) showed that the proportion of *Ascomycota* increased from 22.37% in the CON group to 62.35% in GM, 56.37% in DO, and 59.67% in RR, while the proportion of *Basidiomycota* decreased from 71.53% in the CON group to 32.89% in GM, 35.93% in DO, and 34.59% in RR. At the genus level, the most significant change was the substantial increase in undetected *Monilinia* in CON group to 60.64% in GM, 11.28% in DO, and 0.50% in RR across disease groups (Supplementary Figures S3D,F). When subdivided by leaf and stem organs, leaf analyses revealed that *Rhodotorula* increased in all disease groups (from 35.86% in CON to 42.83% in GM, 60.43% in DO, and 54.90% in RR), with *Monilinia* notably elevated in GM leaves (9.08%). Conversely, *Vishniacozyma* decreased substantially (from 25.15% in CON to 24.54% in GM, 14.20% in DO, and 11.12% in RR) and *Filobasidium* (from 12.19% in CON to 2.76% in GM, 1.73% in DO, and 2.73% in RR) (Supplementary Table S3). Regarding stems, the microbial composition of the GM group differed markedly from those of the other groups. *Monilinia* dominated at 95.01%, while previously dominant leaf taxa such as *Vishniacozyma* and *Filobasidium* were almost undetectable. In the RR and DO groups, *Fusarium* increased (from 5.16% in CON to 10.07% in DO and 8.68% in RR), while *Cadophora* decreased (from 18.19% in CON to 3.59% in DO and 4.82% in RR) (Supplementary Table S4).

### Ecological niche and fungal infectious diseases affect ginseng microbiome diversities

**Figure 1:**
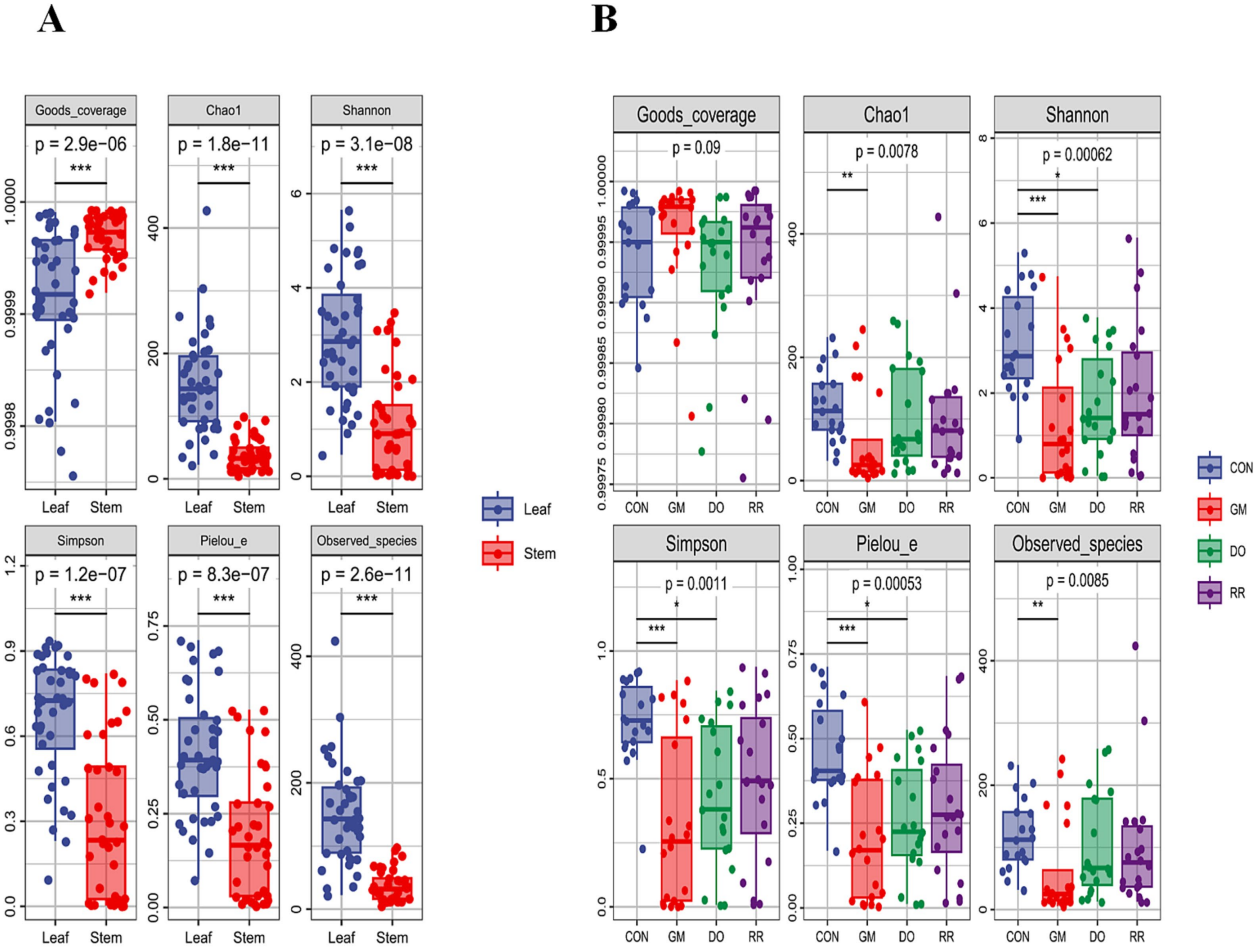
Tissue niche differentiation and fungal pathogen infection shape the α-diversity of the *P. ginseng* phyllosphere microbiota. **(A)** α-Diversity of leaf and stem phyllosphere microbiota in ginseng. **(B)** α-Diversity of phyllosphere microbiota from health control and 3 fungal infectious diseases ginseng.

We compared the α-diversity of leaf and stem microbiomes, finding that leaf microbial richness (Chao1 and Observed species indices), diversity (Shannon and Simpson indices), and evenness (Pielou e) were all significantly higher than those of stems (*p* < 0.01), while Good’s coverage was conversely lower in leaves (Figure 1A). This indicates that despite higher observed levels of α-diversity in leaves, the true diversity is likely to be underestimated due to a larger pool of rare species that are difficult to capture. An overall comparison of α-diversity among infectious disease groups revealed that, under conditions of comparable sampling completeness (i.e., no significant difference in Good’s coverage), diversity indices were reduced to varying degrees in the GM and DO groups, whereas no significant change in α-diversity was observed in the RR group (Figure 1B). When subdividing by ecological niche, analysis of α-diversity in leaves showed no significant differences between the disease groups and the healthy control group (*p* > 0.05). Only marginally higher mean values for diversity and evenness were observed in the CON group compared to the diseased groups (Supplementary Figure S4A). In contrast, significant differences in α-diversity were detected among stem microbiomes (*p* < 0.05). Specifically, the GM stem microbiome exhibited significantly lower species richness, diversity, and evenness, indicating substantial structural alterations in the stem microbiome of *P. ginseng* affected by fungal infection (Supplementary Figure S4B). There were rapid initial increases, followed by an asymptotic approach to plateaus as sequencing depth increased, confirming that the sequencing effort was adequate to capture the overall community composition of the *P. ginseng* microbiome (Supplementary Figure S4C). Further analysis of species accumulation curves indicated the presence of rare species the emerged with increasing sampling effort (Supplementary Figure S4D).

**Figure 2:**
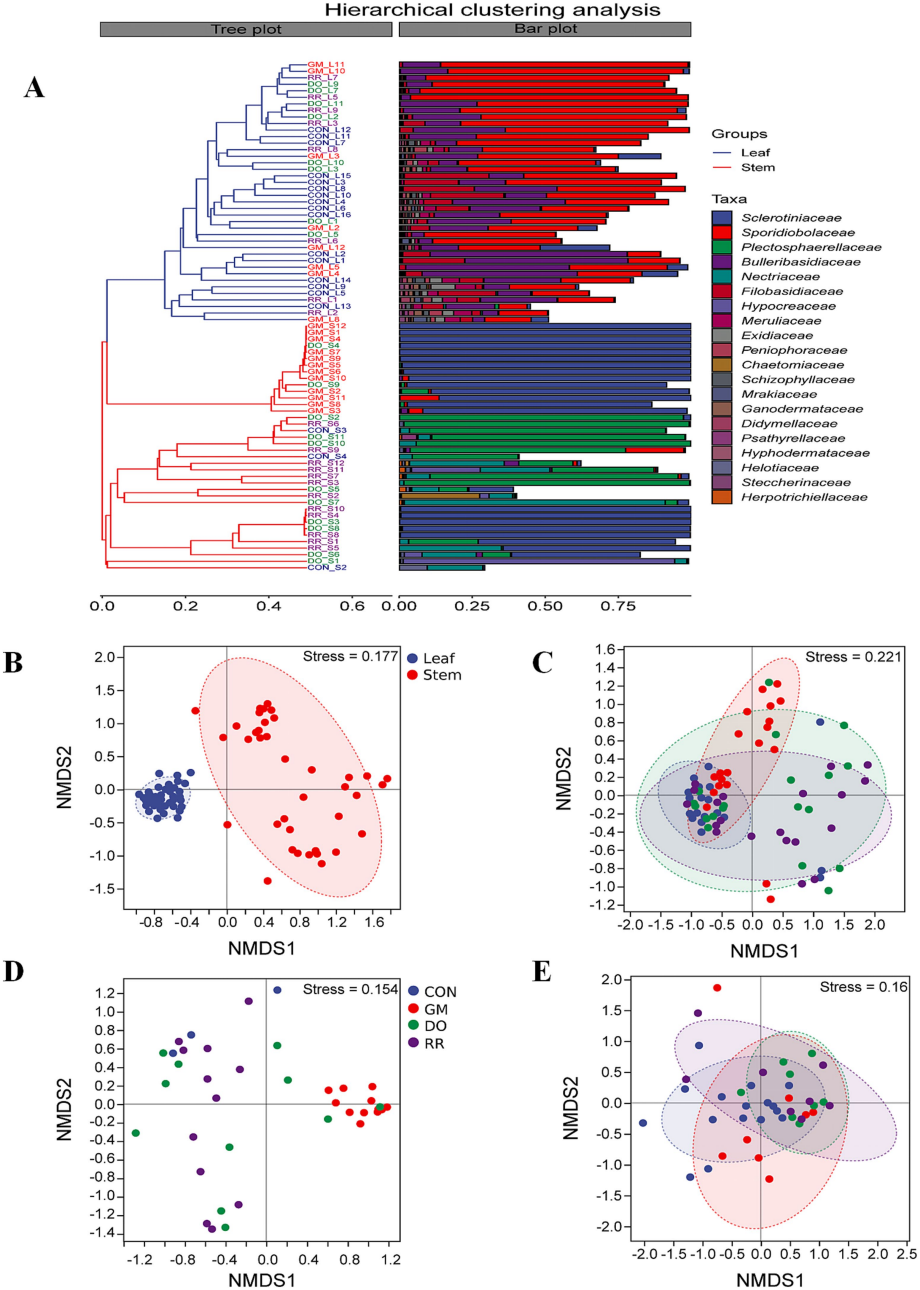
Tissue niche differentiation and fungal pathogen infection shape the community structure (β-diversity) of the *P. ginseng* phyllosphere mycobiome. **(A)** Hierarchical clustering analysis of phyllosphere fungal communities between leaves and stems; NMDS analysis based on Bray-Curtis distances of **(B)** leaves and stems; **(C)** 3 infectious diseases groups and healthy control; **(D)** infectious diseases in leaf **(E)** infectious diseases in stem groups. Between-group differential analysis by PERMANOVA based on Bray-Curtis distances for tissue and disease groups.

**Figure 3:**
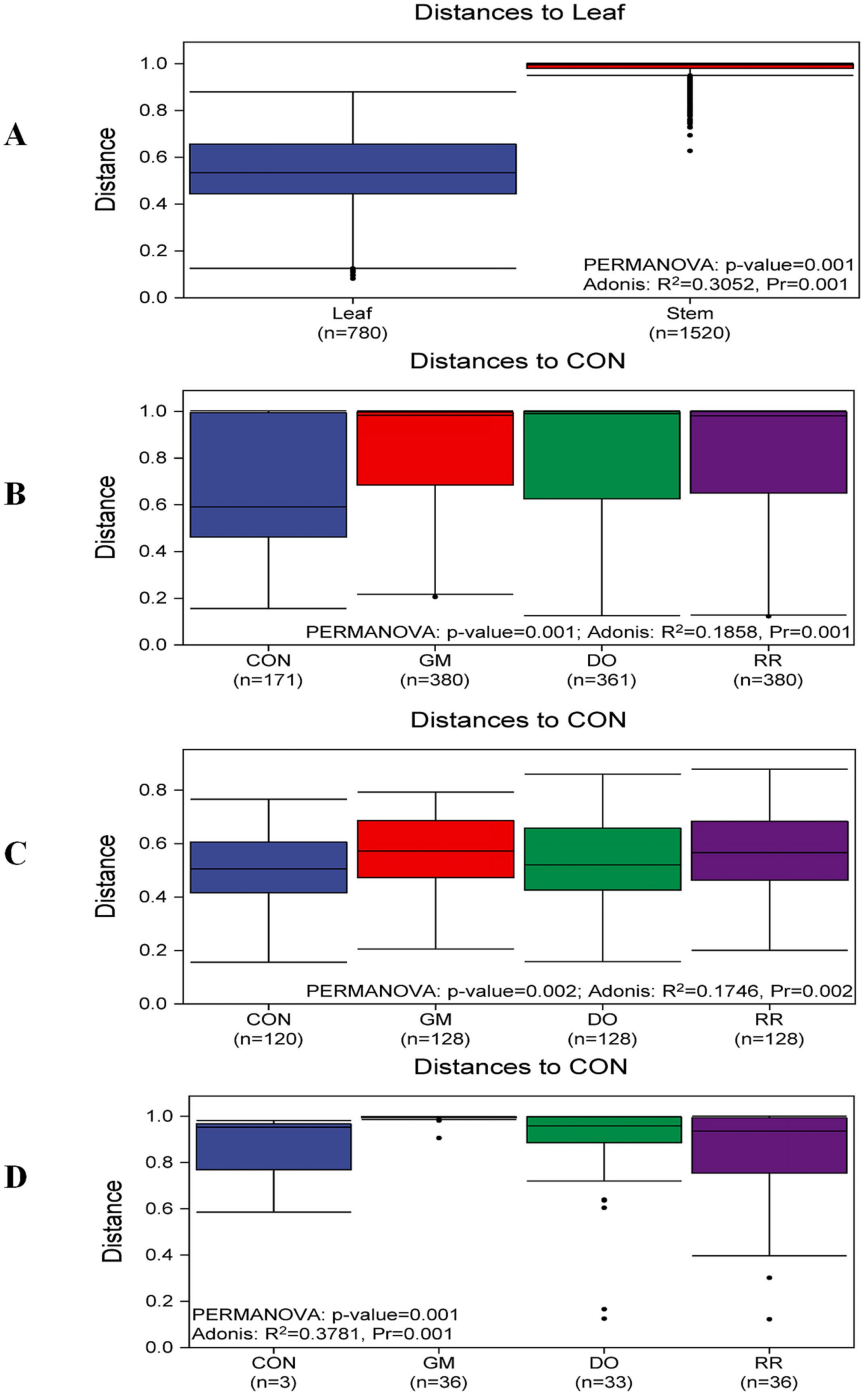
β-Diversity and statistical test of phyllosphere fungal community structure across different groups in *P. ginseng*. **(A)** Leaf and stem **(B)** 3 infectious diseases **(C)** Infectious diseases in leaf **(D)** Infectious diseases in stem groups.

A β-diversity analysis employing UPGMA (Unweighted Pair Group Method with Arithmetic Means) clustering (using the “average” linkage method) on a Bray-Curtis distance matrix, revealed significant differentiation of phyllosphere microbial communities inhabiting between the leaf and stem in *P. ginseng*. Clustering delineated all samples into two major clades: one predominantly comprising leaf samples, and dominated by the families *Sporidiobolaceae* and *Bulleribasidiaceae*; and the other primarily consisting of stem samples, dominated by *Sclerotiniaceae* and *Plectosphaerellaceae* (Figure 2A). This pronounced β-diversity dissimilarity between the phyllosphere microbiota of distinct niches (leaf *vs*. stem) was further corroborated by non-metric multidimensional scaling (NMDS) based on Bray-Curtis distances. NMDS ordination confirmed distinct clustering patterns by organ type (PERMANOVA, *p* = 0.001; Adonis, *R*^2^ = 0.3052, *p* = 0.001), among which leaf samples exhibited tighter clustering (Figure 2B). The NMDS stress value (Stress = 0.177) indicated that the two-dimensional ordination reliably represented the underlying data structure (Supplementary Table S5). Similarly, analysis of β-diversity for the three infectious disease groups, both overall and when subdivided by niche, demonstrated varying degrees of impact on phyllosphere microbial structure compared to the healthy control group (Figures 2C–E, 3).

### Identification of key variables

**Figure 4:**
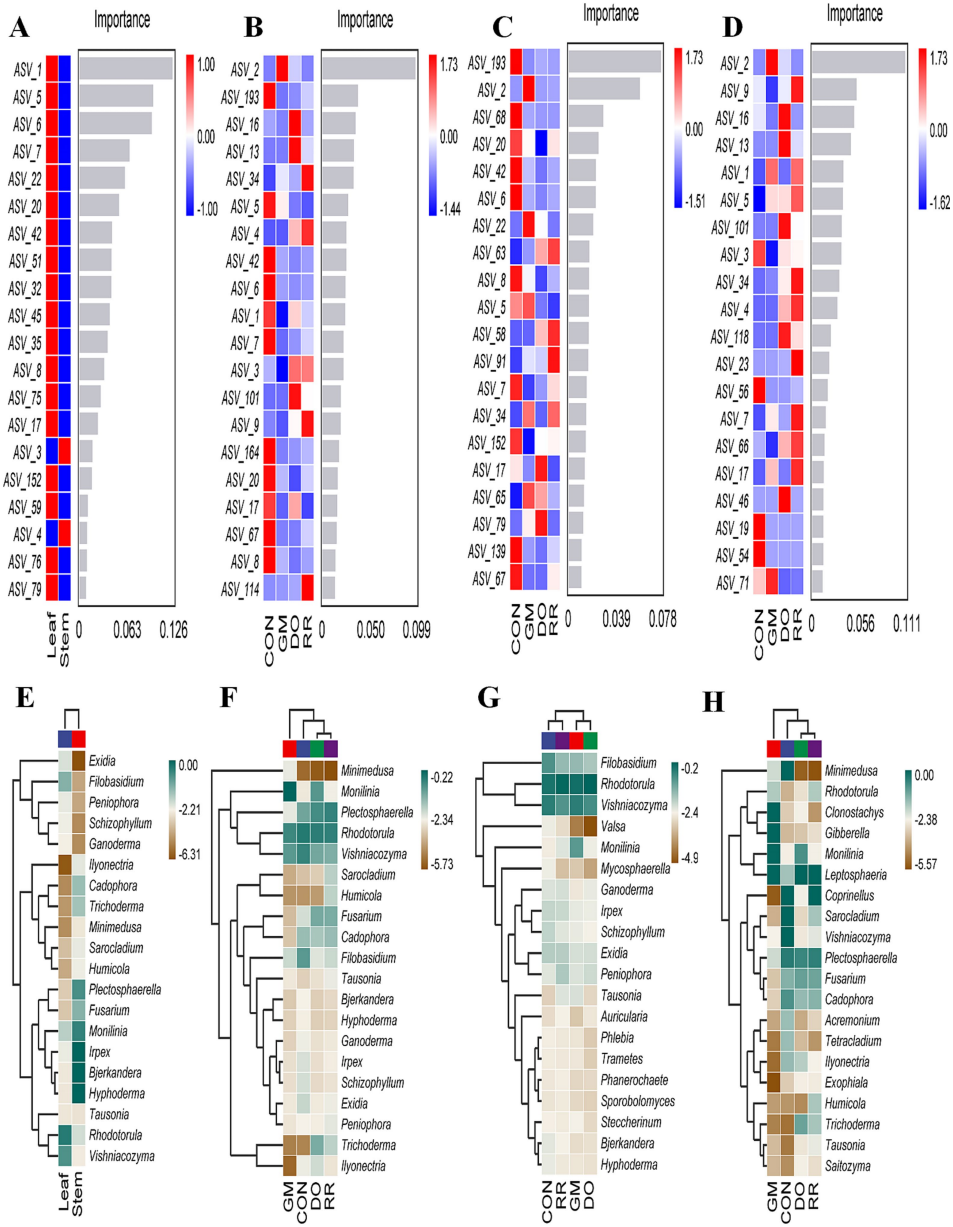
Identification of key microbial taxa in phyllosphere microbiota from different ecological niches and fungal infectious diseases. The potential biomarkers were selected based on the top 20 discriminating features (ASVs) with highest importance scores in the random forest classifier from **(A)** leaf and stem **(B)** 3 infectious diseases **(C)** infectious diseases in leaf **(D)** infectious diseases in stem groups. Heatmap showing relative abundance of the 20 dominant genera in **(E)** leaf and stem **(F)** 3 infectious diseases **(G)** infectious diseases in leaf **(H)** infectious diseases in stem groups.

**Figure 5:**
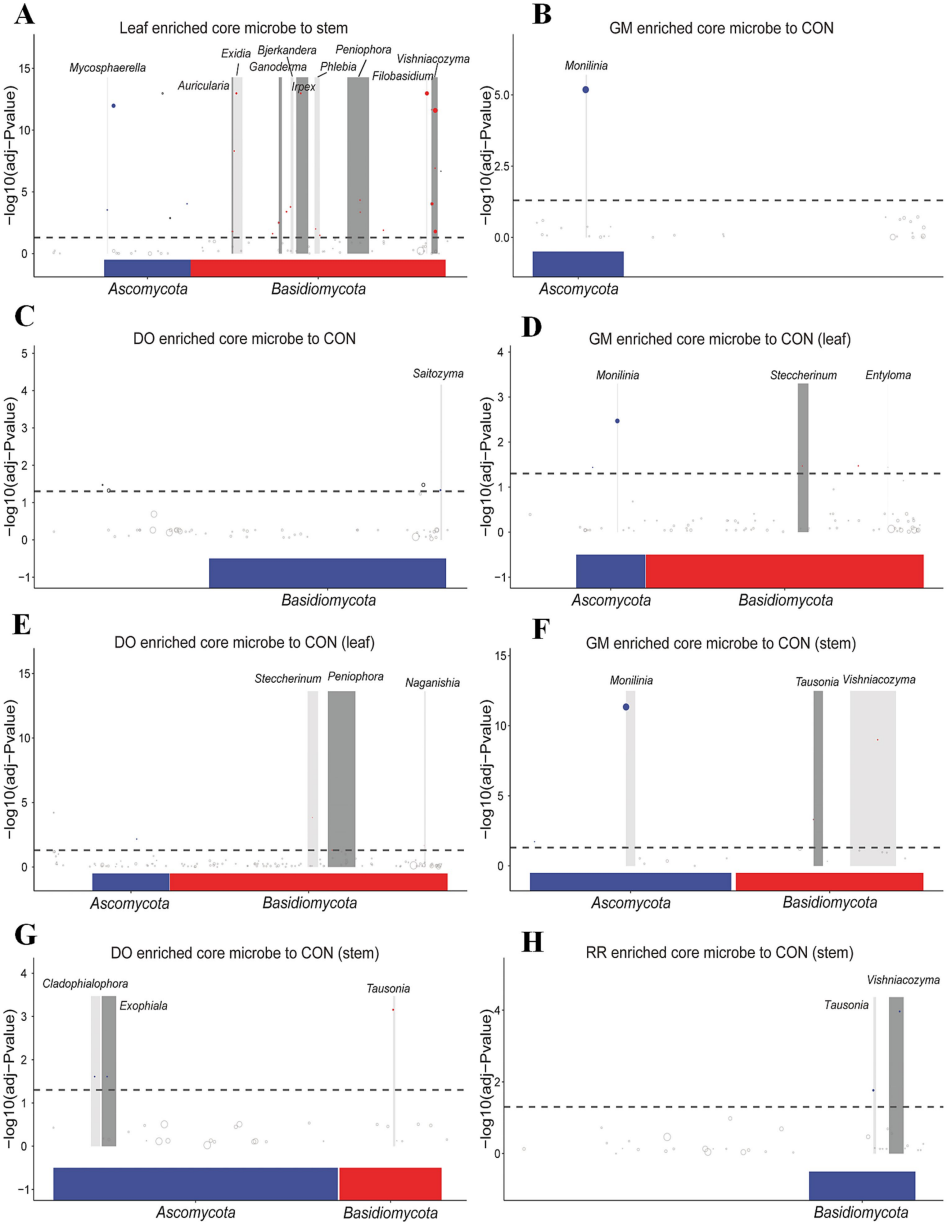
Enriched microbial taxa in the phyllosphere microbiota across different ecological niches and under fungal infectious diseases. **(A)** Leaf and stem **(B)** GM and CON **(C)** DO and CON **(D)** GM and CON in leaf **(E)** DO and CON in leaf **(F)** GM and CON in stem **(G)** DO and CON in stem **(H)** RR and CON in stem groups.

To elucidate the underlying drivers of the observed differences in microbial community composition, we sought to identify the specific taxa whose differential distributions were primarily responsible for these variations. Random Forest (RF), a powerful machine learning algorithm based on decision trees ensembles, is particularly well-suited for analyzing microbial community data, which often exhibits discrete and discontinuous distribution characteristics. We used nested cross-validation with RF analysis, to identify key phyllosphere microbial biomarkers differentiating niches. The 98.72% overall accuracy of the model performance significantly exceeded the 51.28% baseline accuracy (Accuracy Ratio = 1.93). Leaf-associated biomarkers exhibited substantially higher importance values among the top 20 discriminating features (ASVs). These included ASV1 (*Rhodotorula* sp.), ASV5 (*V. victoriae*), ASV6 (*Filobasidium magnum*), ASV7 (*Capnodiales*), ASV22 (*V. victoriae*), ASV20 (*Capnodiales*), ASV42 (*Mycosphaerella tassiana*,), ASV51 (*Exidia glandulosa*), ASV32 (*Exidia japonica*), ASV45 (*Irpex hydnoides*), and ASV35 (*Schizophyllum commune*). Conversely, only two ASVs were enriched in stems among the top 20 ASVs: ASV3 (*Plectosphaerella* sp.) and ASV4 (*Sclerotiniaceae*) (Figure 4A). These findings were corroborated by species composition heatmaps (Figure 4E). Furthermore, to determine whether these differential ASVs exhibited enrichment trends at higher taxonomic levels, we performed pairwise comparisons using the metagenomeSeq method. The results indicated that nine of the top ten microbial taxa enriched in the leaves belonged to the phylum *Basidiomycota* including genera of *Auricularia*, *Exidia*, *Ganoderma*, *Bjerkandera*, *Irpex*, *Phlebia*, *Peniophora*, *Filobasidium*, and *Vishniacozyma*, with the remaining genus *Mycosphaerella* belonging to *Ascomycota* (Figure 5A).

RF analysis of all samples grouped by infectious disease yielded a baseline accuracy of 25.64% and an overall model accuracy of 64.10% (accuracy ratio = 2.50). This modest classification performance aligns with the NMDS analysis of β-diversity (Figure 2C), which indicated significant variation within groups and limited separation between disease groups. Despite this overlap, key biomarker taxa that exhibited significantly increased abundance in each disease group relative to the healthy control group were identified: ASV2 (*M. laxa*, in the GM group); ASV16 (*F. solani*,), ASV13 (*T. hamatum*), ASV4 (*Sclerotiniaceae*), ASV3 (*Plectosphaerella* sp.) and ASV101 (*Exophiala equina*) in the DO group; ASV34 (*Tausonia pullulans*), ASV4 (*Sclerotiniaceae*), ASV3 (*Plectosphaerella* sp.), ASV9 (*Fusarium* sp.), ASV114 (*Holtermanniella takashimae*) in the RR group. Conversely, taxa exhibiting significant decreases included ASV193 (*Mortierella* sp.), ASV5 (*V. victoriae*), ASV42 (*M. tassiana*), ASV6 (*Filobasidium magnum*), ASV1 (*Rhodotorula* sp.), ASV7 (*Capnodiales*), ASV164 (*Malassezia restricta*,), ASV20 (*Capnodiales*), ASV17 (*V. carnescens*), ASV67 (*Valsa mali*,), and ASV8 (*V. victoriae*) (Figures 4B,F,J, 5C). Subdividing by niche, decreased abundance of at least 11 discriminatory biomarkers in disease groups compared to the healthy controls were identified in leaf samples (baseline accuracy = 40.00% *vs*. overall model accuracy = 57.50%, accuracy ratio = 1.44) (Figure 4C). These included ASV193 (*Mortierella* sp.), ASV68 (*Saitozyma podzolica*), ASV20 (*Capnodiales*), ASV42 (*M. tassiana*), ASV6 (*Filobasidium magnum*,), ASV8 (*V. victoriae*), ASV7 (*Capnodiales*), ASV152 (*Taphrina tormentillae*), ASV139 (*Hyphoderma* sp.), and ASV67 (*Valsa mali*). Disease-specific increases in biomarker abundance were also detected: ASV2 (*M. laxa*), ASV22 (*V. victoriae*), ASV5 (*V. victoriae*), ASV34 (*Tausonia pullulans*), and ASV65 (*V. victoriae*) in the GM leaf group; ASV63 (*Peniophora lycii*), ASV58 (*Didymellaceae*), ASV17 (*V. carnescens*), ASV65, ASV79 (*V. carnescens*) in the DO leaf group; ASV63, ASV58, ASV91 (*Sakaguchia* sp., 0.0165), and ASV34 in the RR leaf group. Species composition heatmaps (Figure 4G) and metagenomeSeq analysis (Figures 5B,D,E) further indicated enrichment in diseased leaves relative to controls, notably *Monilinia*, *Steccherinum*, and *Entyloma* in the GM group; *Steccherinum*, *Peniophora*, and *Naganishia* in the DO group. For stems (Figures 4D,H, 5F–H), biomarker analysis revealed distinct enrichment patterns: the GM stem group was characterized by *Monilinia*, *Tausonia*, and *Vishniacozyma*; the DO stem group by *Cladophialophora*, *Exophiala*, and *Tausonia*; and the RR group stem group by *Tausonia* and *Vishniacozyma*.

### Fungal infectious diseases affect ginseng microbiome co-occurrence networks

Network inference analysis was used to identify inherent patterns of co-occurrence or co-exclusion among the microbial members within specific communities, which were driven by spatiotemporal variation and environmental processes. The implementation of Random Matrix Theory (RMT) determined a correlation threshold that effectively separated biologically meaningful interactions from random noise (Supplementary Figures S5A,B). Topological indices at the network and subnetwork levels for different niches are presented in Supplementary Tables S6, S7. The leaf niche had substantially more nodes (143 vs. 41) and edges (174 vs. 72) than the stem niche, suggesting greater microbial diversity and more frequent interactions in the leaf environment. Nevertheless, the phyllosphere microbiota of *P. ginseng* formed a sparse network, evidenced by low overall network density (0.0171 for leaves *vs*. 0.0878 for stems). This suggests that the species relies on a limited number of key interactions. The significantly higher modularity index in leaves (0.8343 vs. 0.5849 in stems) further indicates superior network stability within the leaf niche. Network analysis stratified by infectious disease group (Supplementary Tables S8–S10) revealed sparse networks across all groups (density < 0.1). The healthy group exhibited the highest modularity index (0.8501), indicating greater network stability compared to the three disease groups (GM 0.8456; DO 0.8182; RR 0.8116). Despite having relatively fewer nodes and edges than the other two disease groups, the GM group maintained a relatively high modularity index, indicating preserved network stability and resilience. In contrast, the RR group displayed the lowest modularity index, corresponding to the poorest network stability.

Nodes (ASVs) can be categorized into four distinct topological roles based on their within-module connectivity (*Zi*) and among-module connectivity (*Pi*) values: peripherals (*Zi* < 2.5, *Pi* < 0.62), connectors (*Zi* < 2.5, *Pi* > 0.62), module hubs (*Zi* > 2.5, *Pi* < 0.62), and network hubs (*Zi* > 2.5, *Pi* > 0.62). Peripherals typically represent specialist species with narrow functional niches. Module hubs and connectors represent taxa approaching generalists, while network hubs correspond to super-generalists within the microbial network. The ZIPI analysis of the entire dataset revealed that keystone species were predominantly classified as peripherals (Supplementary Figure S5C), indicating a prevalence of functional specialists. Analysis of the leaf-specific microbial network (Supplementary Figure S5D) identified the genera *Peniophora*, *Vishniacozyma*, *Trametes*, *Phanerochaete*, and *Bjerkandera* exhibiting dual roles as both peripherals and connectors. In contrast, *Pichia*, *Cutaneotrichosporon*, *Irpex*, and *Exidia* were primarily classified as connectors.

### Fungal infectious diseases affect ginseng microbiome function

**Figure 6:**
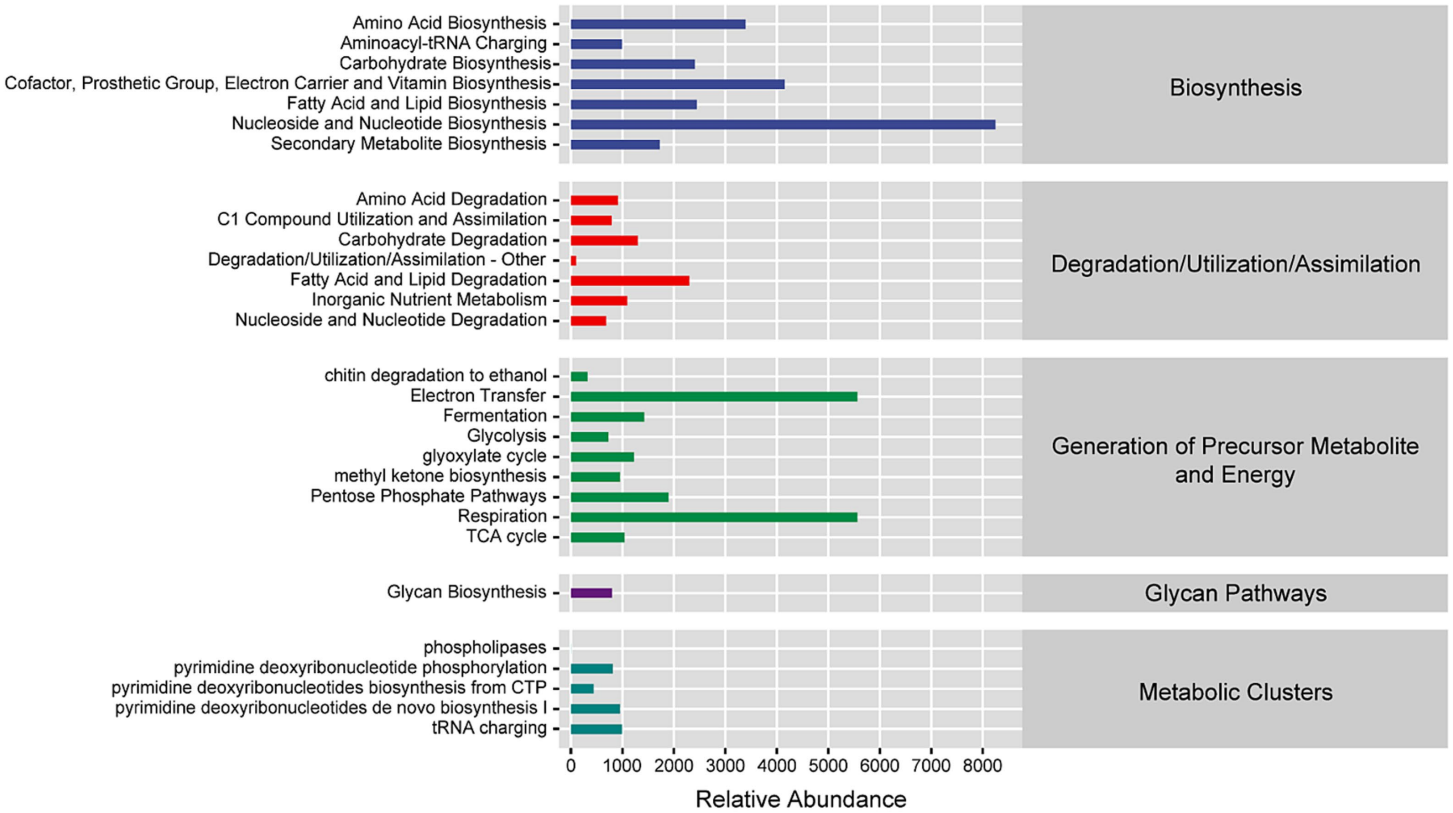
Fungal community functional abundance prediction based on MetaCyc genome database.

**Figure 7:**
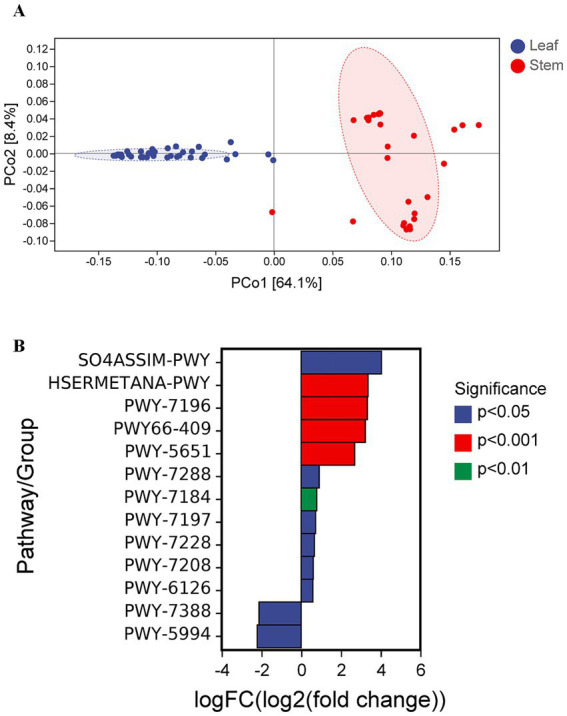
Functional divergence of the fungal community between leaf and stem niches in *P. ginseng* phyllosphere. Low-dimensional ordination of functional profiles via PCoA (Bray-Curtis distance) in **(A)** leaf and stem and **(B)** differential metabolic pathways corresponding to leaf and stem.

While previous analyses have focused on microbial diversity and taxonomic composition, research into microbial ecology also necessitates understanding the functional potential encoded within microbial communities. Predicted functions in the *P. ginseng* phyllosphere microbiota were distributed across biosynthesis, degradation/utilization/assimilation, generation of precursor metabolites and energy, glycan pathways, and metabolic clusters (Figure 6). PCoA based on Bray–Curtis dissimilarity matrices was employed to visualize functional differences in low dimensions. PCoA revealed significant functional divergence between leaf and stem niches (Figure 7A). Using metagenomeSeq, differential abundance analysis identified that the most significantly altered metabolic pathways were upregulated in the leaves compared to the stems. Key upregulated pathways included: SO4ASSIM-PWY [sulfate reduction I (assimilatory)], HSERMETANA-PWY (L-methionine biosynthesis III), PWY-7196 (superpathway of pyrimidine ribonucleoside salvage), PWY66-409 (superpathway of purine nucleotide salvage), and PWY-5651 (L-tryptophan degradation to 2-amino-3-carboxymuconate semialdehyde). The downregulated pathways were primarily PWY-7388 (octanoyl-[acyl-carrier protein] biosynthesis [mitochondria, yeast)] and PWY-5994 [palmitate biosynthesis I (animals and fungi)] (Figure 7B).

**Figure 8:**
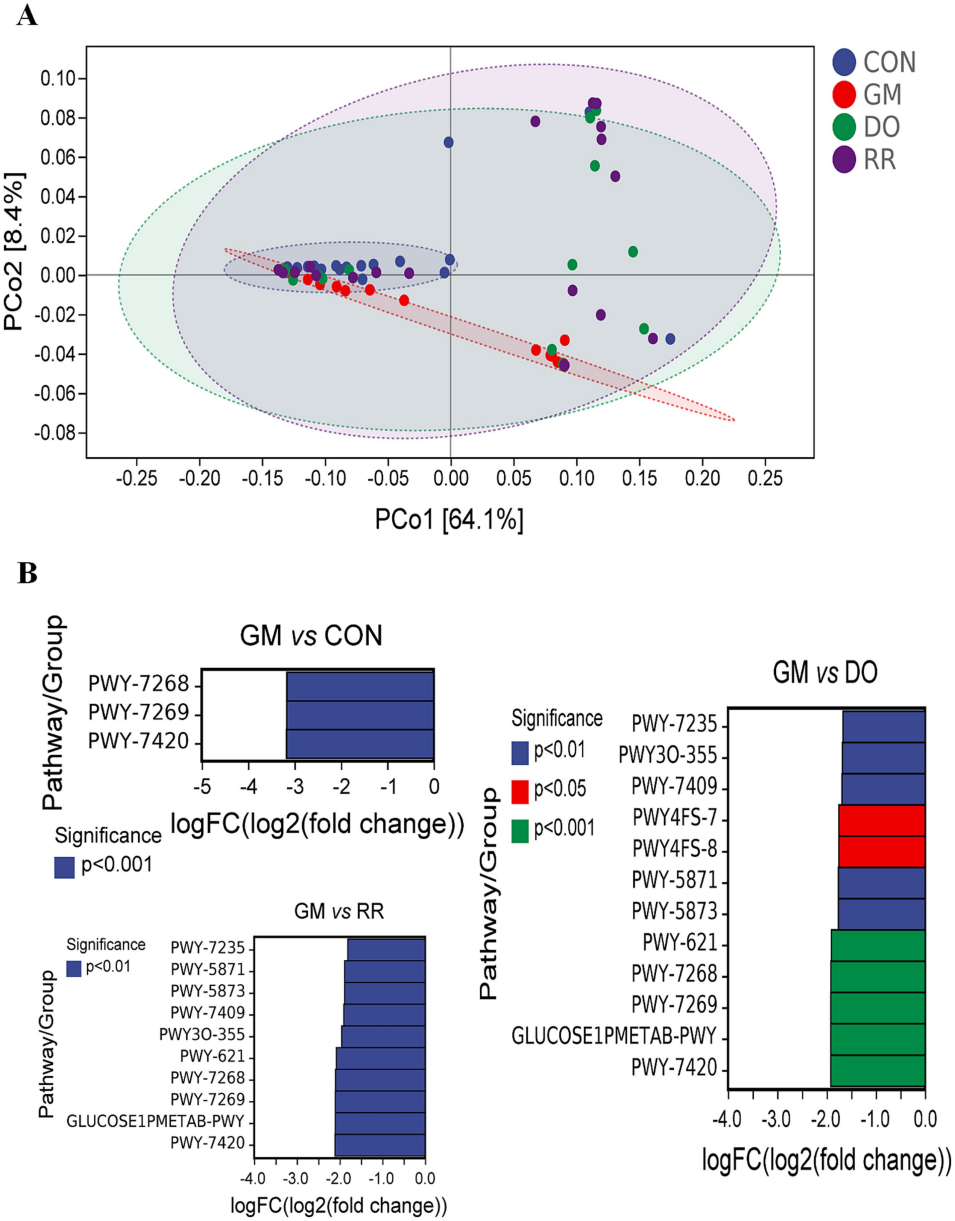
Functional variation of the fungal community across three fungal infectious diseases in the *P. ginseng* phyllosphere. Low-dimensional ordination of functional profiles *via* PCoA (Bray-Curtis distance) in **(A)** 3 infectious diseases **(B)** differential metabolic pathways corresponding to infectious diseases.

**Figure 9:**
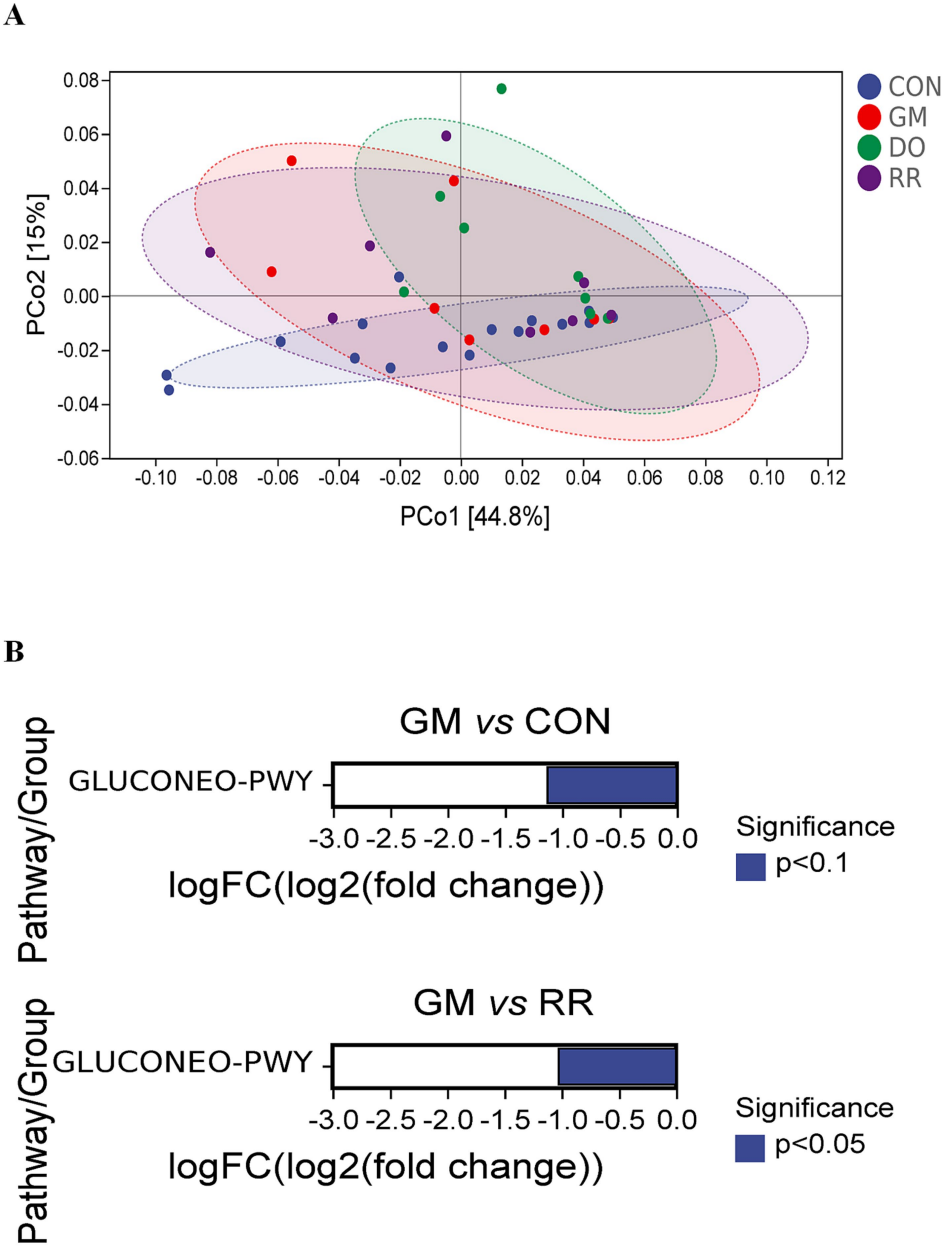
Functional variation of the leaf-associated fungal community across three fungal infectious diseases in *P. ginseng*. Low-dimensional ordination of functional profiles via PCoA (Bray-Curtis distance) in **(A)** infectious diseases in leaf **(B)** differential metabolic pathways corresponding to infectious diseases in leaf.

**Figure 10:**
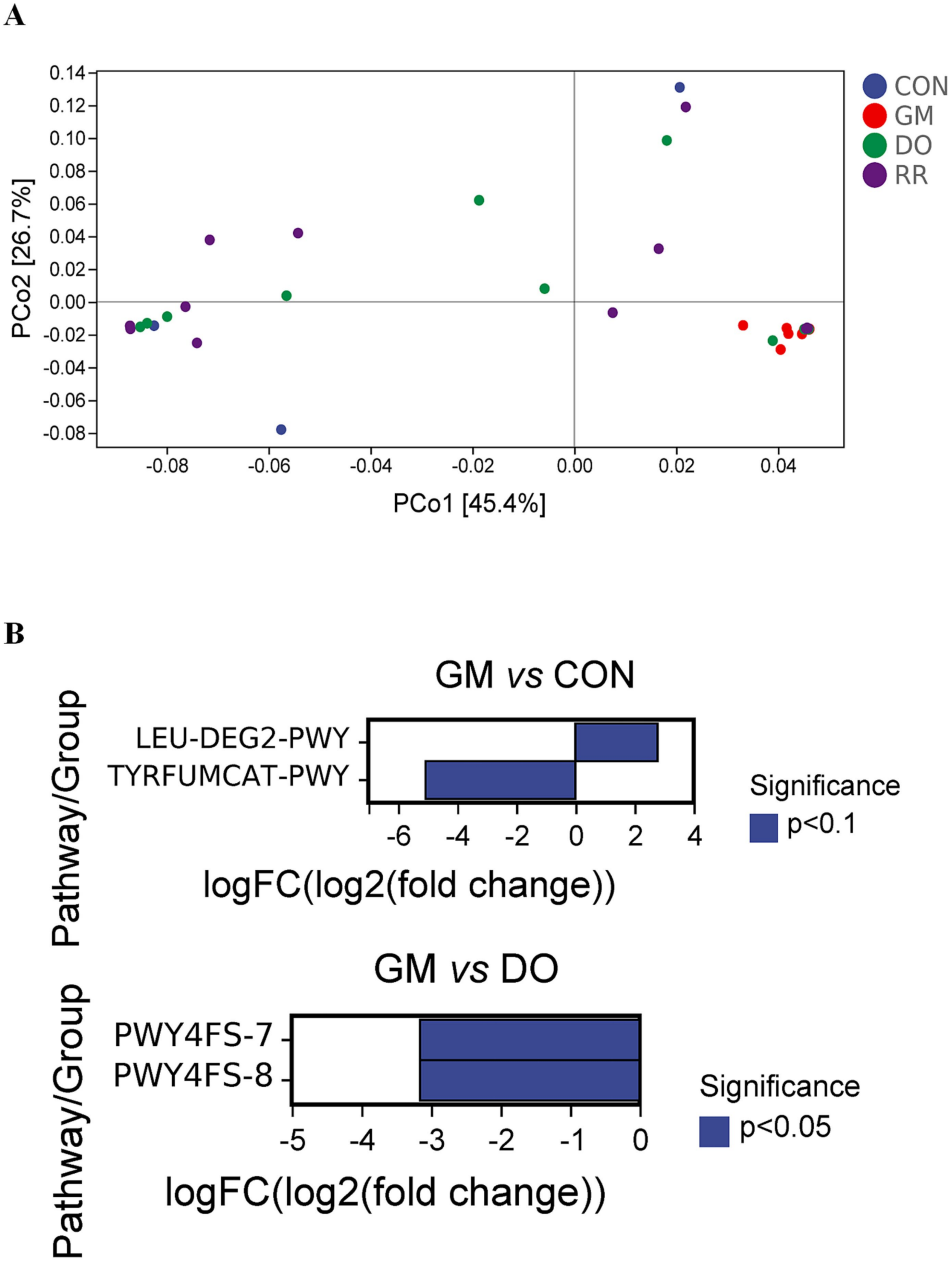
Functional variation of the stem-associated fungal community across three fungal infectious diseases in *P. ginseng*. Low-dimensional ordination of functional profiles via PCoA (Bray-Curtis distance) in **(A)** infectious diseases in stem **(B)** differential metabolic pathways corresponding to infectious diseases in stem.

Significant functional differences were primarily observed between the GM group and the healthy control (CON) group, or other disease groups (DO, RR), both overall and within specific niches, due to high within-group dispersion. In the overall analysis, compared to CON group, GM exhibited significant downregulation of PWY-7268 [NAD/NADP-NADH/NADPH cytosolic interconversion (yeast)], PWY-7269 [NAD/NADP-NADH/NADPH mitochondrial interconversion (yeast)], PWY-7420 [monoacylglycerol metabolism (yeast)] (Figures 8A,B). Compared to RR, GM significantly downregulated 10 pathways: PWY-7235 [superpathway of ubiquinol-6 biosynthesis (eukaryotic)], PWY-5871 [ubiquinol-9 biosynthesis (eukaryotic)], PWY-5873 [ubiquinol-7 biosynthesis (eukaryotic)], PWY-7409 [phospholipid remodeling (phosphatidylethanolamine, yeast)], PWY3O-355 [stearate biosynthesis III (fungi)], PWY-621 [sucrose degradation III (sucrose invertase)], PWY-7268 [NAD/NADP-NADH/NADPH cytosolic interconversion (yeast)], PWY-7269 [NAD/NADP-NADH/NADPH mitochondrial interconversion (yeast)], GLUCOSE1PMETAB-PWY (glucose and glucose-1-phosphate degradation), PWY-7420 [monoacylglycerol metabolism (yeast)]. Additionally, compared to RR, GM downregulated 2 phosphatidylglycerol biosynthesis pathways: PWY4FS-7 [phosphatidylglycerol biosynthesis I (plastidic)] and PWY4FS-8 [phosphatidylglycerol biosynthesis II (non-plastidic)]. Analysis stratified by niche (leaf and stem, Figures 9A, 10A) confirmed the predominant pattern of significant pathway downregulation in the GM group, including GLUCONEO-PWY (gluconeogenesis I), LEU-DEG2-PWY (L-leucine degradation III), TYRFUMCAT-PWY (L-tyrosine degradation I), and PWY4FS-7/8 (phosphatidylglycerol biosynthesis I/II) (Figures 9B, 10B).

## Discussion

*Botrytis cinerea* (gray mold) and *Monilinia* spp. (brown rot) are common fungal pathogens that share overlapping infection sites and a requirement on high humidity (>80% RH). They also exhibit similar early-stage symptoms (e.g., fruit browning), which often leads to misdiagnosis in the field. Specifically, field observations initially identified *P. ginseng* samples (GM group) as gray mold, however, amplicon sequencing revealed significant enrichment of *Monilinia*—particularly in stems (95.01% relative abundance) and no significant *B. cinerea* detection. This finding is consistent with emerging reports of *Monilinia*-induced ginseng brown rot (Bai et al., 2000; Bai, 2002), underscoring the necessity of integrating sporulation patterns (cottony gray *vs*. concentric rings), host tropism (rosaceous bias), and conidial morphology (clustered *vs*. chain-forming spores) for accurate field diagnosis.

Our results revealed significant shifts in the abundance of key fungal taxa under disease stress. Genera such as *Monilinia* and *Fusarium*—well-documented pathogens responsible for brown rot and root rot in *P. ginseng*, respectively—were markedly increased. Conversely, taxa with recognized antagonistic potential, such as *Trichoderma* and *Rhodotorula*, showed variable responses. Notably, beneficial epiphytic yeast genera like *Vishniacozyma* and *Filobasidium*, which contribute to phyllosphere microbiome stability, decreased in diseased leaves. These shifts collectively indicate a pathogen-dominated restructuring of the phyllosphere community, accompanied by a limited alteration in potentially protective taxa. Notably, the pathogen profiles that showed significant abundance increases in this study were not perfectly aligned with the primary pathogens traditionally reported for each corresponding disease in the field. This apparent discrepancy can be interpreted through an ecological perspective of the phyllosphere microbiome: First, disease may be driven by a pathogenic consortium, in which a dominant pathogen (e.g., *M. laxa* in the GM group) alters the host microenvironment and facilitates the co-proliferation of opportunistic pathogens (e.g., *Fusarium* spp.) (Vannier et al., 2019). Second, many of the detected pathogens are facultative saprophytes whose pathogenicity is condition-dependent; their increased abundance is likely a consequence rather than the cause of infection, emerging after host tissue damage (Trivedi et al., 2020). Furthermore, DNA-based abundance does not equate to pathogenic activity, as part of the signal may arise from saprophytic growth following tissue necrosis (Blazewicz et al., 2013). Thus, plant health depends on the overall balance of the microbial community. Our findings support a strategic shift from single-pathogen control toward holistic microbiome management aimed at enhancing community resilience.

Fan et al. (2022a, 2022b) summarized and found that the cultivation-dependent analyses of *P. ginseng* endophytes reveal substantial taxonomic diversity and tissue-specific colonization patterns. Different ecological niches of plants (e.g., leaves vs. stems) significantly influence their community composition through differences in anatomical structure, microenvironment, and resource allocation. Their functional diversity is directly linked to plant health and ecosystem stability (Xiong et al., 2021a; Xiong et al., 2021b). The fungal community in the phyllosphere is taxonomically bifurcated into yeasts and filamentous fungi (Andrews and Harris, 2000). On healthy plant leaves, although bacteria numerically dominate the phyllosphere microbiota, yeasts and yeast-like fungi (e.g., *Aureobasidium pullulans*) exhibit significant ecological activity as primary epiphytic colonizers (Andrews and Harris, 2000). These yeasts belong to both Basidiomycota and Ascomycota, with Basidiomycota-dominated groups (*Cryptococcus*, *Rhodotorula*, and *Sporobolomyces*) being most prevalent (Nakase et al., 2005; SlÁviková et al., 2009). While Ascomycota-associated yeasts include *Debaryomyces hansenii*, *Hanseniaspora uvarum*, *Kazachstania barnetii*, *Metschnikowia pulcherrima*, *Metschnikowia reukaufii*, *Pichia membranifaciens*, *Saccharomyces cerevisiae*, and multiple *Candida* spp. (Glushakova and Chernov, 2010; Limtong and Koowadjanakul, 2012). Detected in both healthy controls and diseased samples in this study, these yeasts may play stabilizing roles in maintaining phyllosphere microbial structure and pathogen resistance. The loss of beneficial yeast functions may stem from fundamental alterations in the ecological niche under disease stress. Pathogen invasion triggers the plant’s basal immune response (PTI), leading to reactive oxygen species (ROS) bursts and the synthesis of defensive metabolites (Jones et al., 2024). While this antimicrobial environment aims to suppress pathogens, it can also impose nonspecific stress on a broad spectrum of microorganisms, including potential beneficial microbes, thereby reshaping the microbial community (Yu et al., 2022). The widespread downregulation of pathways related to energy metabolism and membrane lipid synthesis observed in this study may reflect the plant’s reallocation of resources toward core defense mechanisms under stress, resulting in reduced metabolic investment in maintaining complex symbiotic networks (Hacquard and Schadt, 2015).

Filamentous fungi are frequently implicated as phytopathogens and are a key focus of phyllosphere research (Keen, 2000). Their population density in healthy leaves typically ranges from 10^2^ to 10^8^ CFU/g, with the fungi predominantly existing as dormant spores rather than active mycelia (Whipps et al., 2008). In order to survive the fluctuating phyllosphere conditions (e.g., temperature, humidity, light), these fungi must evade abiotic/biotic stresses before they can initiate infections. In the current study, the abundance of pathogenic fungi notably increased in diseased stems. The phyllosphere harbors diverse filamentous fungi, including *Cladosporium*, *Alternaria*, *Fusarium*, *Penicillium*, *Acremonium*, *Colletotrichum*, *Mucor*, and *Aspergillus*, representing both pathogenic and non-pathogenic taxa (Inácio et al., 2002; Abdelfattah et al., 2015). Phyllosphere microbes are integral to plant health, with pathogenic strains causing diseases, and non-pathogenic consortia contributing to disease suppression (Balint-Kurti et al., 2010). This study further revealed that pathogen infection not only alters species composition but also disrupts the interaction network structure of the microbial community. Highly modular and densely interconnected microbial networks are recognized as indicators of community stability and functional resilience (Faust and Raes, 2012). The observed decline in network stability under disease conditions reflects weakened synergistic interactions among species, which may directly compromise the “collective immunity” of the community—its ability to suppress pathogen invasion through mechanisms such as nutrient competition and spatial niche occupation. For instance, phyllosphere yeasts have been demonstrated to directly inhibit the growth of pathogenic fungi via strategies such as siderophore competition (Innerebner et al., 2011).

The dynamics of phyllosphere microbes vary across plant developmental stages. Abdelfattah et al. (2015) documented the highest fungal diversity in olive leaves compared to flowers and fruits, with *Colletotrichum* (a pathogen) peaking in mature fruits. While our study sampled plants at a uniform growth stage, intra-group variability likely reflects asynchronous disease progression among individuals. Recent studies highlight plants’ capacity to modulate phyllosphere microbiota homeostasis. For example, Chen et al. (2020) found that the wild-type *Arabidopsis thaliana* (Col-0) maintains phyllosphere diversity through PTI pathways, MIN7 vesicle trafficking, and CAD1 gene regulation, in contrast, mutant (*mfec*) leaves exhibit Proteobacteria overgrowth and Firmicutes suppression. In our study, pathogen infection reduced phyllosphere fungal diversity, though bacterial responses remain to be explored. Notably, such microbiome disturbances are not limited to the aerial parts of the plant. In the rhizosphere, continuous cropping of *P. ginseng* leads to similar dysbiosis, exacerbating soil-borne diseases and severely limiting plant growth and survival, a phenomenon known as “continuous cropping obstacle” (Chen et al., 2025). The aforementioned findings reveal the interconnected dysbiosis between the aboveground and belowground microbiomes of plants, suggesting that any effective microecological regulation strategy must adopt a whole-plant systemic perspective. Based on this, our study provides clear directions for the development of next-generation green control technologies: First, targeted screening of probiotics should be directly based on the key beneficial taxa identified in healthy plants in this study (e.g., *V. victoriae*), with attempts to construct synthetic microbial communities capable of restoring stable network functions (Sharma and Araujo, 2025). Second, phyllosphere microbiome engineering can be achieved by developing foliar probiotic formulations based on nanocarriers or biofilms, with application timing aimed at preemptively establishing beneficial microbial dominance before disease onset. Finally, agronomic management strategies must adopt a dual approach: regulating the field microenvironment and improving soil health. This integrated strategy will systematically enhance the microbiome resilience and disease resistance of the entire plant. Ultimately, our objective is to shift from the control of “single pathogens” toward the scientific management of the overall health of the “plant-microbiome holobiont,” which represents a critical step toward achieving sustainable agriculture.

## Conclusion

While culture-independent sequencing reveals the taxonomic profiles of the phyllosphere, functional exploration requires complementary, cultivation-based approaches due to the prevalence of unculturable taxa. Our study reveals that fungal infections in *P. ginseng* reduce microbial diversity, alter community structure, destabilize interaction networks, and downregulate key metabolic pathways. Although beneficial yeasts like *Rhodotorula* increased in leaves under disease stress, pathogens such as *M. laxa* ultimately dominated, especially in stems. Further work should integrate multi-omics (holo-omics) to link taxonomic shifts with functional dynamics during plant-microbe-pathogen interactions. This will help elucidate how microbial gene expression and metabolic activity respond to infection (Ouyang et al., 2024; Ge and Wang, 2025). These findings provide a foundation for sustainable disease management strategies in ginseng cultivation: constructing synthetic communities derived from native beneficial taxa, developing phyllosphere-targeted probiotic formulations, and integrating agronomic practices to holistically enhance the resilience of the plant-microbiome system, thereby advancing toward ecological and systemic crop protection.

## Data Availability

The datasets presented in this study can be found in online repositories. The names of the repository/repositories and accession number(s) can be found below: https://www.ncbi.nlm.nih.gov/, PRJNA1291187.

## Footnotes

1
https://docs.qiime2.org/2019.4/tutorials/

2
https://metacyc.org/

3
https://www.kegg.jp/
