# Supplementary material for: Disease-induced changes in Panax ginseng phyllosphere fungal community assembly and functional adaptation
Source: Front Microbiol. 2026 Mar 26;17:1740520. doi: 10.3389/fmicb.2026.1740520 (PMC13062315; doi:10.3389/fmicb.2026.1740520)
Supplement: Supplementary file 1 [file Data_sheet_1.docx]

# SUPPLEMENTARY MATERIAL

# Disease-induced changes in Panax ginseng phyllosphere fungal community assembly and functional adaptation

## Supplementary Tables

**Table S1 The average sequence count per sample after raw sequences underwent quality control, denoising, merging, and chimera removal processes.**

| Sample | Input | Filtered | Denoised | Merged | Non#chimeric | Non#singleton |
| --- | --- | --- | --- | --- | --- | --- |
| Total | 119992±14224 | 119466±14220 | 119014±14161 | 102990±17535 | 102090±17137 | 102090±17137 |
| Leaf | 127995±9952 | 127484±9999 | 126935±10078 | 101697±20495 | 100670±19888 | 100670±19889 |
| Stem | 109936±12317 | 109393±12233 | 109063±12178 | 104615±12982 | 103874±12916 | 103874±12916 |
| GM | 118681±14409 | 118078±14329 | 117596±14158 | 106685±15941 | 105732±15551 | 105732±15551^a^ |
| DO | 115306±13209 | 114764±13151 | 114268±12971 | 102648±15928 | 101592±15453 | 101592±15453^a^ |
| RR | 116894±12608 | 116302±12508 | 115795±12346 | 107657±14010 | 106972±13955 | 106972±13955^b^ |
| CON | 130125±12637 | 129783±12714 | 129478±12857 | 93800±21713 | 92898±20956 | 92897±20956^a^ |
| GM-L | 125510±9399 | 124876±9379 | 124197±9324 | 103509±17038 | 101812±15996 | 101812±15996 |
| DO-L | 123811±11883 | 123213±11838 | 122476±11807 | 101350±21430 | 100601±20936 | 100601±20936 |
| RR-L | 125068±9019 | 124417±8936 | 123741±8909 | 108644±18034 | 108018±18017 | 108018±18017 |
| CON-L | 134564±6301 | 134292±6290 | 134050±6269 | 96235±23732 | 95269±22869 | 95268±22869 |
| GM-S | 111853±15623 | 111281±15508 | 110994±15398 | 109861±14800 | 109652±14705 | 109652±14705^b^ |
| DO-S | 106800±8128 | 106315±8123 | 106061±8106 | 103946±8356 | 102583±7811 | 102583±7811^b^ |
| RR-S | 110083±11206 | 109540±11119 | 109174±10992 | 106834±10344 | 106100±10216 | 106100±10216^b^ |
| CON-S | 112368±16952 | 111748±16848 | 111189±17118 | 84059±3402 | 83414±4065 | 83414±4064^a^ |

**Table S2 Statistical table of microbial taxonomic unit counts at the species level in two organs (leaves and stems).**

| ID | Leaf | Stem |
| --- | --- | --- |
| *Monilinia laxa* | 0.021294 | 0.357282 |
| *Vishniacozyma victoriae* | 0.176659 | 0.003096 |
| *Filobasidium magnum* | 0.06292 | 7.12E-05 |
| *Trichoderma hamatum* | 6.51E-06 | 0.027128 |
| *Cadophora orchidicola* | 5.39E-06 | 0.02536 |
| *Fusarium solani* | 0.000345 | 0.023004 |
| *Vishniacozyma carnescens* | 0.020444 | 0.000458 |
| *Cadophora luteo-olivacea* | 9.94E-06 | 0.014868 |
| *Plectosphaerella oratosquillae* | 0.000197 | 0.008536 |
| *Sarocladium strictum* | 0.000273 | 0.00822 |
| Others | 0.717846 | 0.531976 |

**Supplementary** Table S3

**Supplementary Table S3 Statistical table of microbial taxonomic unit counts at the genus level in leaves under different pathogen infections.**

| ID | CON | GM | DO | RR |
| --- | --- | --- | --- | --- |
| *Rhodotorula* | 0.358608 | 0.428262 | 0.604323 | 0.549023 |
| *Vishniacozyma* | 0.251514 | 0.245431 | 0.141968 | 0.111185 |
| *Filobasidium* | 0.121862 | 0.027602 | 0.017272 | 0.027284 |
| *Monilinia* | 0.002884 | 0.090775 | 0.004085 | 0.005842 |
| *Exidia* | 0.014035 | 0.006896 | 0.007467 | 0.013445 |
| *Peniophora* | 0.005472 | 0.006182 | 0.008432 | 0.016148 |
| *Irpex* | 0.01016 | 0.00484 | 0.004337 | 0.009084 |
| *Schizophyllum* | 0.010711 | 0.004562 | 0.003435 | 0.005551 |
| *Ganoderma* | 0.006267 | 0.002382 | 0.004676 | 0.00712 |
| *Tausonia* | 0.00109 | 0.006538 | 0.001345 | 0.005877 |
| Others | 0.217397 | 0.176529 | 0.20266 | 0.249441 |

**Table S4 Statistical table of microbial taxonomic unit counts at the genus level in stems under different pathogen infections.**

| ID | CON | GM | DO | RR |
| --- | --- | --- | --- | --- |
| *Monilinia* | 0.003696 | 0.9501 | 0.19191 | 0.004453 |
| *Plectosphaerella* | 0.41304 | 0.010329 | 0.275612 | 0.360431 |
| *Cadophora* | 0.18192 | 0.000872 | 0.035868 | 0.048218 |
| *Fusarium* | 0.051579 | 0.000552 | 0.100698 | 0.086819 |
| *Trichoderma* | 1.22E-05 | 2.53E-05 | 0.090815 | 0.023263 |
| *Ilyonectria* | 0.034751 | 9.12E-06 | 0.016883 | 0.004135 |
| *Rhodotorula* | 0.00023 | 0.019573 | 0.001898 | 0.01974 |
| *Tetracladium* | 0.032076 | 1.76E-05 | 0.000335 | 6.42E-05 |
| *Acremonium* | 0.030684 | 0.000136 | 0.00016 | 0.001044 |
| *Leptosphaeria* | 0.026331 | 0 | 0 | 0 |
| Others | 0.225682 | 0.018387 | 0.285821 | 0.451833 |

**Table S5 Intergroup difference analysis based on Bray-Curtis distance permutation test statistics (all with 999 permutations).**

| **Groups** | **PERMANOVA** | | **Adonis** | |
| --- | --- | --- | --- | --- |
|  | **p-value (all)** | **p-value (*vs*)** | **R^2^** | **Pr (>F)** |
| **Organs** | 0.001 | Leaf *vs* Stem: 0.001 | 0.305181 | 0.001 |
| **Diseases** | 0.001 | CON *vs* GM: 0.001  CON *vs* DO: 0.004  CON *vs* RR: 0.002  GM *vs* DO: 0.001  GM *vs* RR: 0.001  DO *vs* RR: 0.655 | 0.185834 | 0.001 |
| **Diseases-leaf** | 0.002 | CON *vs* GM: 0.011  CON *vs* DO: 0.002  CON *vs* RR: 0.019  GM *vs* DO: 0.051  GM *vs* RR: 0.139  DO *vs* RR: 0.71 | 0.174594 | 0.002 |
| **Diseases-stem** | 0.001 | CON *vs* GM: 0.002  CON *vs* DO: 0.341  CON *vs* RR: 0.229  GM *vs* DO: 0.001  GM *vs* RR: 0.001  DO *vs* RR: 0.386 | 0.378095 | 0.001 |

**Table S6 The network-level topological indices for different niche/disease groups.**

| **topo** | **empirical_network** | **random_network_avg** | **random_network_sd** |
| --- | --- | --- | --- |
| **Average_nearest_neighbor_degree** | 3.6751 | 3.7844 | 0.1239 |
| **Average_path_length** | 6.3412 | 4.7375 | 0.0972 |
| **Betweenness_centrality** | 173801 | 182304.2 | 37781.07 |
| **Closeness_centrality** | 0.5046 | 1.6001 | 0.3298 |
| **Degree_assortativity** | 0.4429 | -0.0172 | 0.0697 |
| **Degree_centralization** | 1272 | 788.4 | 200.7227 |
| **Density** | 0.0184 | 0.0184 | 0 |
| **Cluster_num** | 10 | 9.6 | 1.9551 |
| **Diameter** | 9.3943 | 10.8 | 0.7888 |
| **Transitivity** | 0.3487 | 0.0147 | 0.0078 |
| **Num_vertice** | 156 | 156 | 0 |
| **Num_edge** | 222 | 222 | 0 |
| **Modularity** | 0.8304 | 0.6017 | 0.0173 |

**Table S7 The subnetwork-level topological indices for different niches groups.**

| **Group** | **Average nearest neighbor degree** | **Average path length** | **Betweenness centrality** | **Closeness centrality** | **Degree assortativity** | **Degree centralization** | **Density** | **Cluster num** | **Diameter** | **Transitivity** | **Num vertice** | **Num edge** | **Modularity** |
| --- | --- | --- | --- | --- | --- | --- | --- | --- | --- | --- | --- | --- | --- |
| **Leaf** | 3.0713 | 6.6317 | 158066.8 | 0.5933 | 0.125 | 653 | 0.0171 | 12 | 9.3943 | 0.2727 | 143 | 174 | 0.8343 |
| **Stem** | 4.7952 | 2.9796 | 3225 | 0.7113 | 0.3375 | 307 | 0.0878 | 6 | 4.087 | 0.4368 | 41 | 72 | 0.5849 |

**Table S8 The subnetwork-level topological indices for different disease groups.**

| **Group** | **Average nearest neighbor degree** | **Average path length** | **Betweenness centrality** | **Closeness centrality** | **Degree assortativity** | **Degree centralization** | **Density** | **Cluster num** | **Diameter** | **Transitivity** | **Num vertice** | **Num edge** | **Modularity** |
| --- | --- | --- | --- | --- | --- | --- | --- | --- | --- | --- | --- | --- | --- |
| **CON** | 2.9462 | 6.4298 | 94650 | 0.4113 | 0.2517 | 555 | 0.0198 | 17 | 7.6493 | 0.3546 | 119 | 139 | 0.8501 |
| **GM** | 2.8665 | 5.7301 | 36457 | 0.425 | 0.1336 | 338 | 0.025 | 10 | 7.3892 | 0.2953 | 89 | 98 | 0.8456 |
| **DO** | 3.6289 | 6.7511 | 138880 | 0.5457 | 0.3964 | 910 | 0.0222 | 8 | 9.4936 | 0.3514 | 126 | 175 | 0.8182 |
| **RR** | 3.7512 | 6.4389 | 138138.5 | 0.5769 | 0.4503 | 1054 | 0.0224 | 8 | 9.3943 | 0.3642 | 130 | 188 | 0.8116 |

**Table S9 The network-level topological indices in leaves for different disease groups**

| **topo** | **empirical_network** | **random_network_avg** | **random_network_sd** |
| --- | --- | --- | --- |
| **Average_nearest_neighbor_degree** | 26.7031 | 26.5971 | 0.1394 |
| **Average_path_length** | 1.952 | 1.7496 | 0.0005 |
| **Betweenness_centrality** | 134947.8 | 4634.089 | 1362.198 |
| **Closeness_centrality** | 10.9112 | 4.2325 | 1.3027 |
| **Degree_assortativity** | 0.2735 | -0.0166 | 0.0259 |
| **Degree_centralization** | 1678 | 1241.2 | 345.9726 |
| **Density** | 0.2511 | 0.2511 | 0 |
| **Cluster_num** | 1 | 1 | 0 |
| **Diameter** | 2.2304 | 2.9 | 0.3162 |
| **Transitivity** | 0.5452 | 0.2513 | 0.0031 |
| **Num_vertice** | 104 | 104 | 0 |
| **Num_edge** | 1345 | 1345 | 0 |
| **Modularity** | 0.5885 | 0.1391 | 0.006 |

**Table S10 The subnetwork-level topological indices in leaves for different disease groups.**

| **Group** | **Average nearest neighbor degree** | **Average path length** | **Betweenness centrality** | **Closeness centrality** | **Degree assortativity** | **Degree centralization** | **Density** | **Cluster num** | **Diameter** | **Transitivity** | **Num vertice** | **Num edge** | **Modularity** |
| --- | --- | --- | --- | --- | --- | --- | --- | --- | --- | --- | --- | --- | --- |
| **GM** | 21.0323 | 1.7018 | 5976.442 | 6.2913 | 0.0159 | 654 | 0.3665 | 1 | 1.9692 | 0.6229 | 57 | 585 | 0.5217 |
| **DO** | 22.5589 | 1.8095 | 5317.011 | 4.8958 | 0.4323 | 528 | 0.3629 | 1 | 2.5579 | 0.675 | 59 | 621 | 0.4719 |
| **RR** | 12.5017 | 2.0444 | 19787.15 | 9.5889 | 0.3041 | 640 | 0.2686 | 1 | 2.4678 | 0.7867 | 46 | 278 | 0.5477 |

##
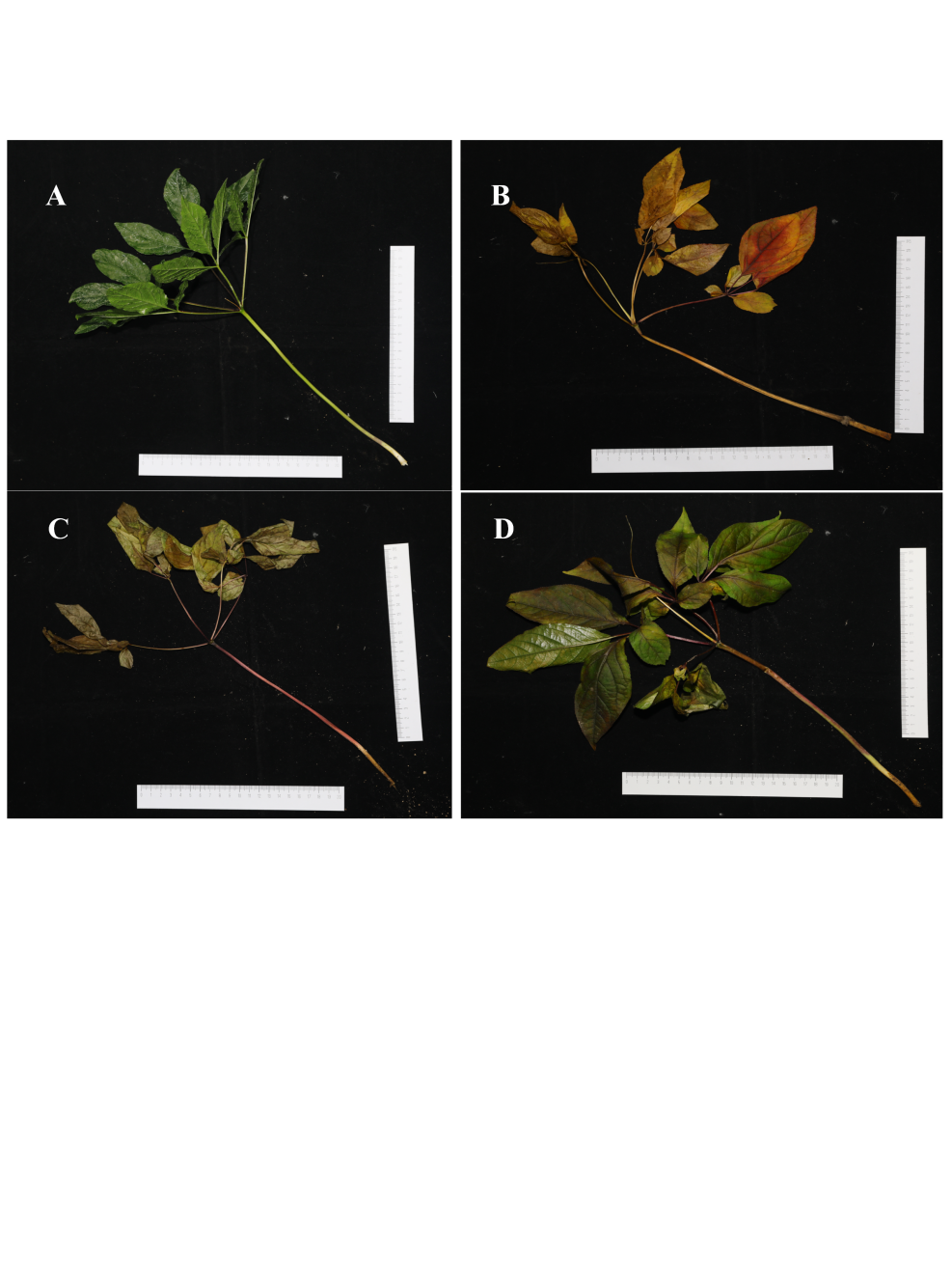
Supplementary Figures

**Figure S1 Representative symptom images of gray mold (GM), damping-off (DO), root rot (RR) and healthy control (CON) groups samples.** (A) Healthy control; (B) Gray mold, typically appears as watery, brownish lesions on leaves and stems, often covered with a characteristic grayish, fluffy mold under humid conditions. The infected tissues become soft and necrotic, leading to leaf blight, stem wilting, or collapse; (C) Damping-off, mainly affects seedlings and the lower stem near the soil line. Symptoms include water-soaked, dark brown to black lesions at the stem base, causing seedlings to collapse ("damp off"). In older plants, it can cause stem girdling, wilting, and stunted growth; (D) Root rot, the symptoms are primarily belowground. Infected roots turn brown to black, become soft and decayed, and often show reduced root mass. Aboveground symptoms include yellowing of leaves, stunting, and gradual wilting—especially under water stress—due to impaired root function.

**
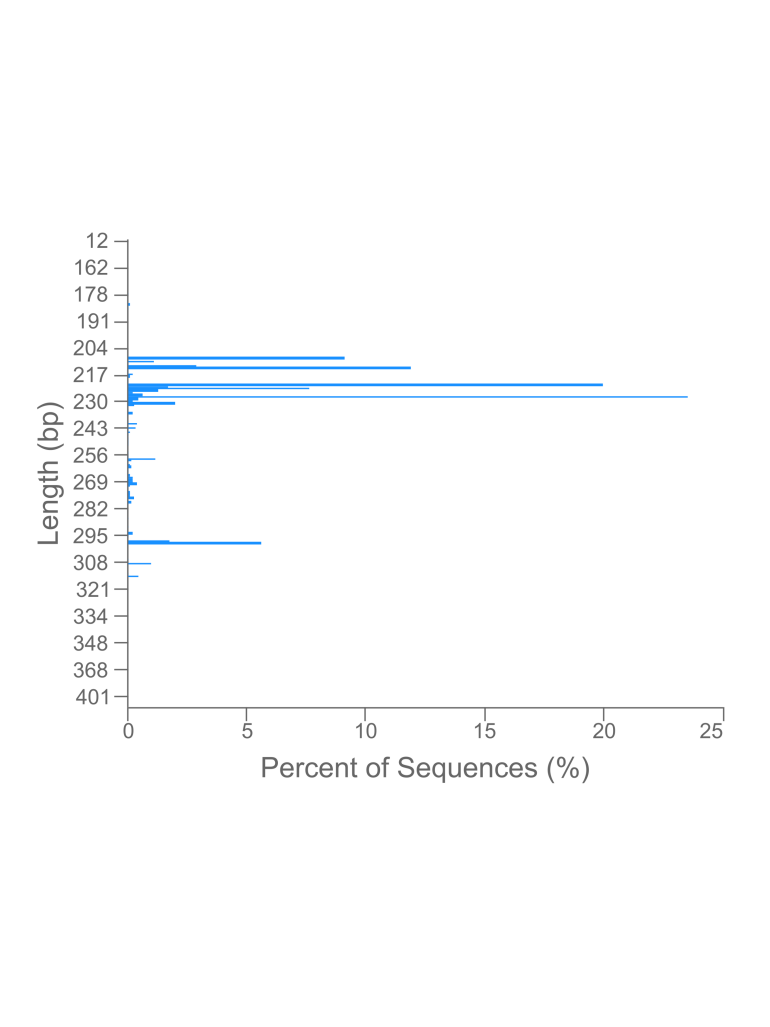
Figure S2 The percentage of sequences length distribution.**


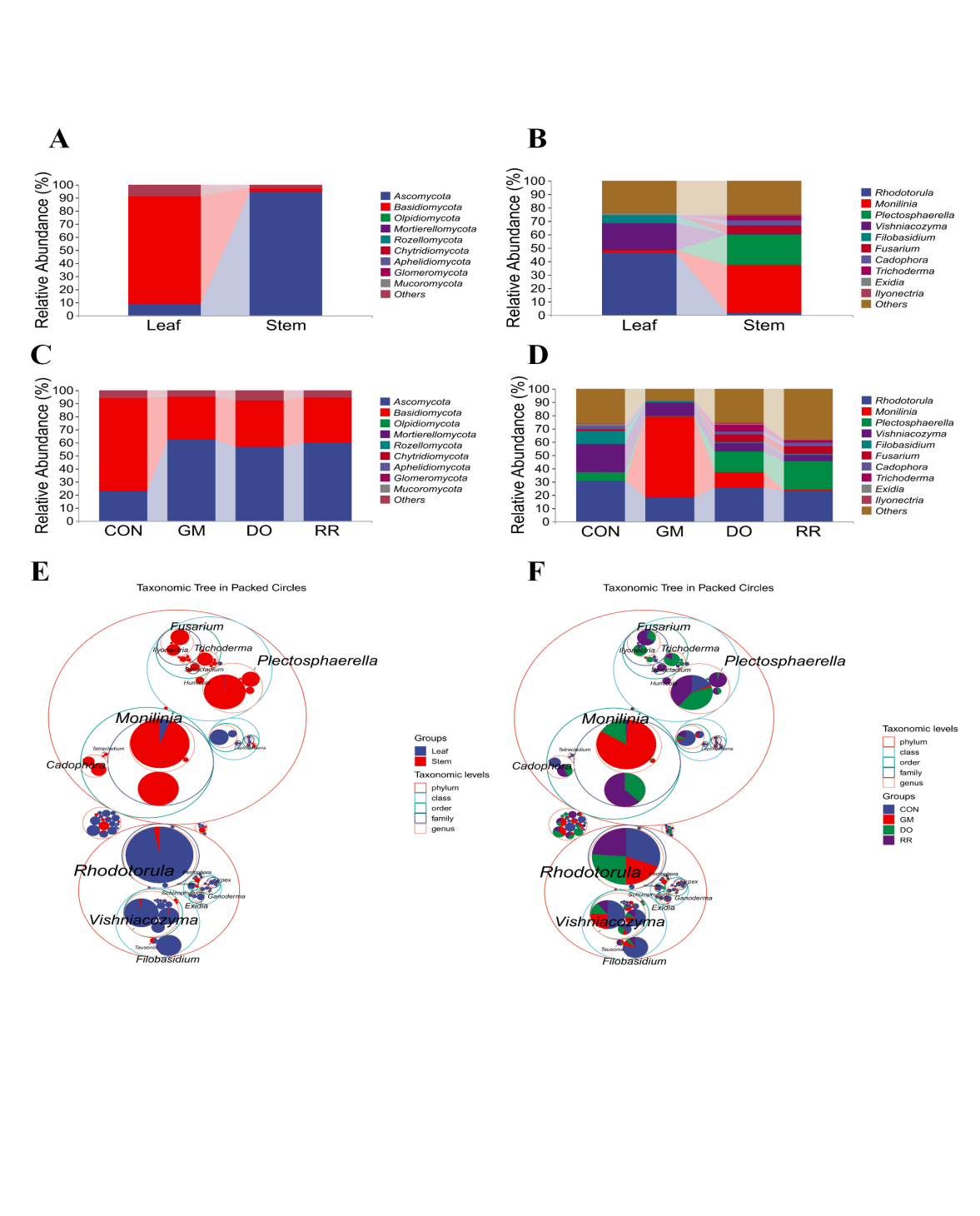
**Figure S3 Taxonomic composition of fungal communities.** (A) Phylum-level composition across tissue types; (B) Genus-level composition across tissue types; (C) Phylum-level composition under distinct infectious diseases; (D) Genus-level composition under distinct infectious diseases; (E) Phylogenetic tree of dominant fungal nera (tissue-specific); (F) Phylogenetic tree of dominant fungal genera (disease-specific).


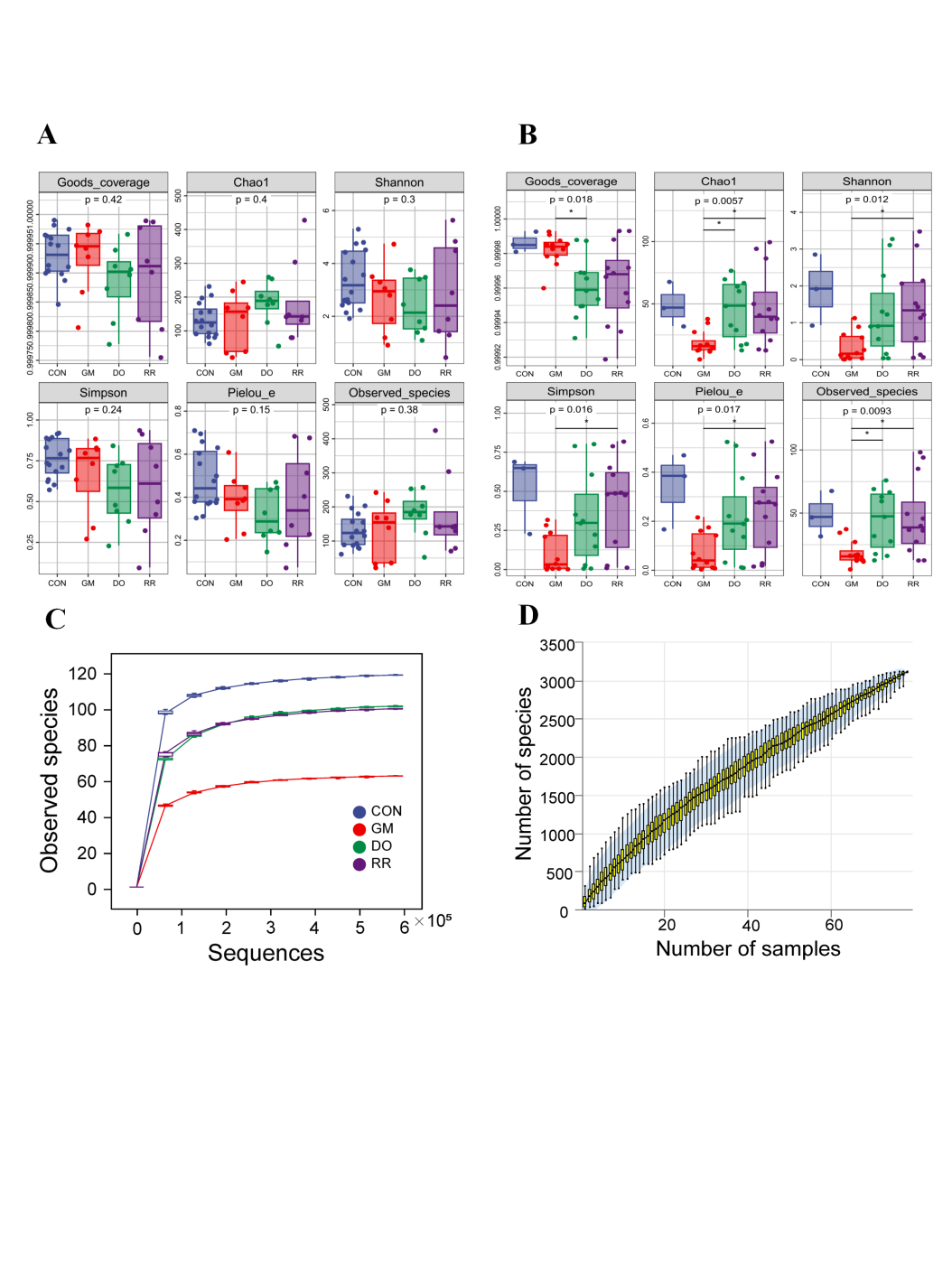
**Figure S4 The α diversity analysis.** (A) The α diversity of leaf tissues under different infectious diseases; (B) The α diversity of stem tissues under different infectious diseases; (C) Rarefaction curve; (D) Species accumulation curve.


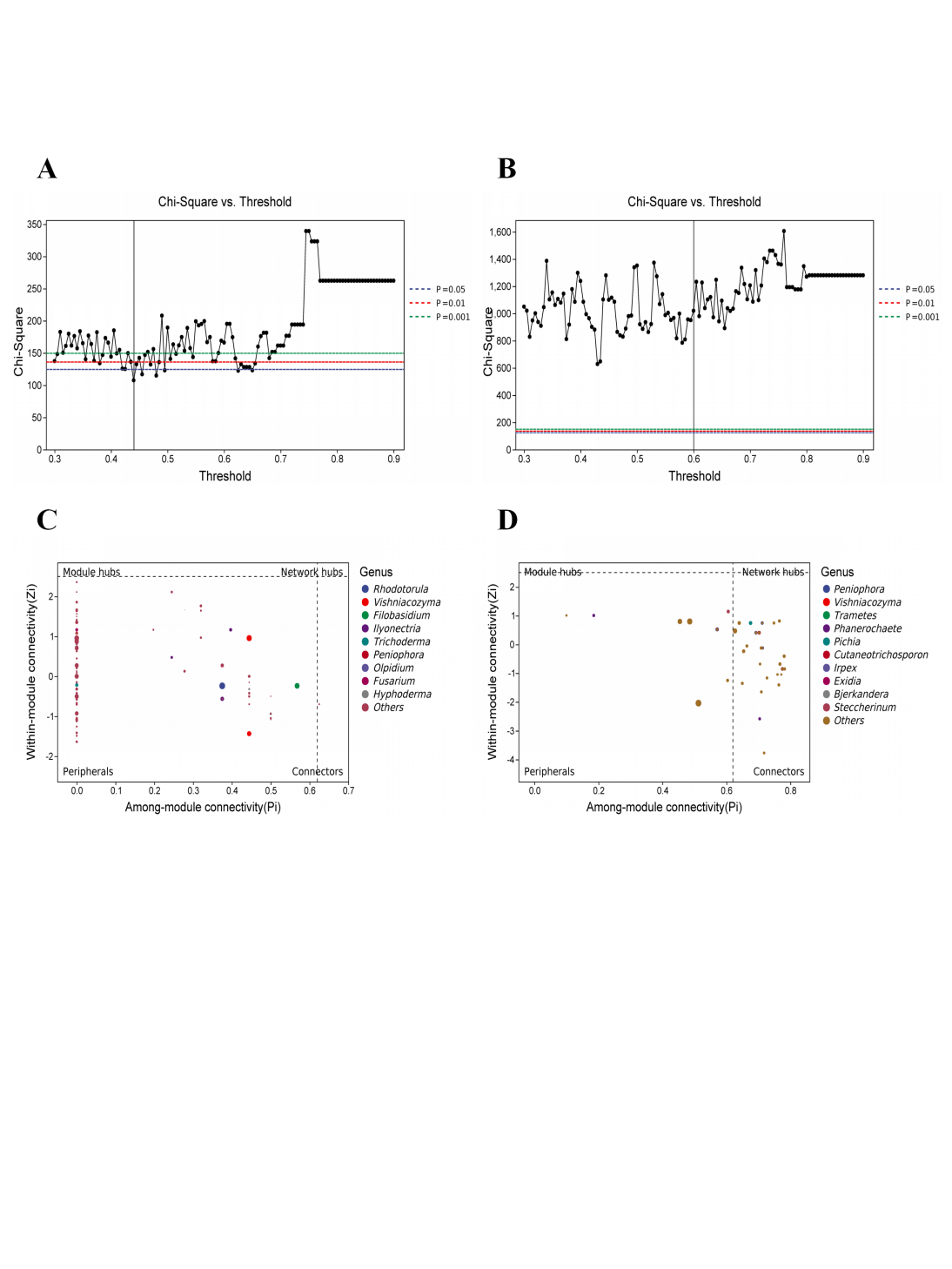
**Figure S5 The implementation of Random Matrix Theory (RMT) determined a correlation threshold that effectively separated biologically meaningful interactions from random noise.**
